# The Effect of Intermittent Antenatal Iron Supplementation on Maternal and Infant Outcomes in Rural Viet Nam: A Cluster Randomised Trial

**DOI:** 10.1371/journal.pmed.1001470

**Published:** 2013-06-18

**Authors:** Sarah Hanieh, Tran T. Ha, Julie A. Simpson, Gerard J. Casey, Nguyen C. Khuong, Dang D. Thoang, Tran T. Thuy, Sant-Rayn Pasricha, Thach D. Tran, Tran Tuan, Terence Dwyer, Jane Fisher, Beverley-Ann Biggs

**Affiliations:** 1 Department of Medicine, University of Melbourne, Royal Melbourne Hospital, Parkville, Victoria, Australia; 2 Research and Training Centre for Community Development, Hanoi, Viet Nam; 3 Centre for Molecular, Environmental, Genetic and Analytic Epidemiology, Melbourne School of Population and Global Health, University of Melbourne, Melbourne, Victoria, Australia; 4 Provincial Centre of Preventive Medicine, Ha Nam Province, Viet Nam; 5 The Jean Hailes Research Unit, School of Public Health and Preventive Medicine, Monash University, Victoria, Australia; 6 Centre for Women's Health Gender and Society, Melbourne School of Population and Global Health, University of Melbourne, Parkville, Victoria, Australia; 7 Murdoch Childrens Research Institute, The Royal Children's Hospital, Parkville Victoria, Australia; 8 The Victorian Infectious Diseases Service, Royal Melbourne Hospital, Parkville Victoria, Australia; Epicentre, France

## Abstract

**Background:**

Anemia affects over 500 million women, and in pregnancy is associated with impaired maternal and infant outcomes. Intermittent antenatal iron supplementation is an attractive alternative to daily dosing; however, the impact of this strategy on infant outcomes remains unclear. We compared the effect of intermittent antenatal iron supplementation with daily iron supplementation on maternal and infant outcomes in rural Viet Nam.

**Methods and Findings:**

This cluster randomised trial was conducted in Ha Nam province, Viet Nam. 1,258 pregnant women (<16 wk gestation) in 104 communes were assigned to daily iron–folic acid (IFA), twice weekly IFA, or twice weekly multiple micronutrient (MMN) supplementation. Primary outcome was birth weight. Mean birth weight was 3,148 g (standard deviation 416). There was no difference in the birth weights of infants of women receiving twice weekly IFA compared to daily IFA (mean difference [MD] 28 g; 95% CI −22 to 78), or twice weekly MMN compared to daily IFA (MD −36.8 g; 95% CI −82 to 8.2). At 32 wk gestation, maternal ferritin was lower in women receiving twice weekly IFA compared to daily IFA (geometric mean ratio 0.73; 95% CI 0.67 to 0.80), and in women receiving twice weekly MMN compared to daily IFA (geometric mean ratio 0.62; 95% CI 0.57 to 0.68), but there was no difference in hemoglobin levels. Infants of mothers who received twice weekly IFA had higher cognitive scores at 6 mo of age compared to those who received daily IFA (MD 1.89; 95% CI 0.23 to 3.56).

**Conclusions:**

Twice weekly antenatal IFA or MMN did not produce a clinically important difference in birth weight, when compared to daily IFA supplementation. The significant improvement in infant cognitive outcomes at 6 mo of age following twice weekly antenatal IFA requires further exploration, and provides additional support for the use of intermittent, rather than daily, antenatal IFA in populations with low rates of iron deficiency.

**Trial registration:**

Australia New Zealand Clinical Trials Registry 12610000944033

*Please see later in the article for the Editors' Summary*

## Abstract 2

Beverley-Anne Biggs and colleagues conduct a community-based cluster randomized trial in rural Viet Nam to compare the effect of antenatal iron-folic acid supplementation taken daily or twice weekly on maternal and infant outcomes.

*Please see later in the article for the Editors' Summary*

## Editors' Summary

**Background:**

Anemia is a common condition in which the blood does not supply the body with enough oxygen because of a low number of red blood cells or low levels of hemoglobin—the iron-containing pigment that enables red blood cells to carry oxygen. Iron deficiency is the most common cause of anemia worldwide and, according to the World Health Organization, affects over 2 billion people: half of all pregnant women and 40% of preschool children in low- and middle-income countries are thought to be anemic. Anemia contributes to 20% of all maternal deaths and is also linked to increased maternal morbidity, higher rates of preterm birth and low birth weight, and reduced infant survival, with potential long-term consequences for child growth and development. Identifying and treating iron deficiency anemia is therefore a global health priority.

**Why Was This Study Done?:**

Daily iron–folic acid supplementation given from early in pregnancy is the standard recommended approach to prevent and treat anemia in pregnant women, but recently the World Health Organization recommended intermittent use because of poor compliance with daily regimes (because of side effects) and poor bowel absorption. However, the evidence from many of the studies used to support this recommendation was of poor quality, and so it remains unclear whether intermittent supplementation is as, or more, effective than daily supplementation, especially in lower income settings where antenatal testing for anemia is not readily available. So in this study, the researchers conducted a community-based cluster randomized trial (where groups of people are randomized, rather than individuals) in rural Viet Nam to compare the effect of antenatal iron–folic acid supplementation taken twice weekly (either alone, or in combination with other micronutrients) with daily iron–folic acid supplementation, on maternal and infant outcomes during the first six months of life.

**What Did the Researchers Do and Find?:**

The researchers randomized 104 communes in Ha Nam Province, Viet Nam, and enrolled 1,258 women who were less than 16 weeks pregnant into the study between September and November 2010. Although the researchers intended to register the trial before the study started, registration was delayed by a month because the supplements arrived earlier than the researchers anticipated, and they thought it best to start recruiting at that time to avoid the Vietnamese New Year, when women might be travelling. Each woman was interviewed and had blood taken for hemoglobin and iron indices (ferritin) before receiving daily iron–folic acid supplementation (426 women), twice weekly iron–folic acid supplementation (425 women), or twice weekly iron–folic acid supplementation plus micronutrients (407 women). The women had follow-up assessments at 32 weeks gestation, delivery, and at six months postpartum: their infants were assessed at birth and at six months old.

The researchers found that at enrollment, the women's average hemoglobin concentration was 123 g/l, and 12.6% of the women were anemic. At 32 weeks gestation, 10.8% of the women were anemic, but there was no difference in hemoglobin levels between the three supplement groups. The average ferritin level was 75.6 µg/l at enrollment, with 2.2% of women iron deficient. Ferritin levels decreased from enrollment to 32 weeks gestation in all supplement groups but were lower in women who took twice weekly supplements. The researchers also found that birth weight (the primary outcome) was similar in all supplement groups, and there were also no differences in gestational age or in the risk of prematurity, stillbirth, or early neonatal death. At six months, there were also no differences in the levels of infant hemoglobin, prevalence of anemia, or growth rates. However, infants born to mothers in the twice weekly iron–folic acid group had improved cognitive development compared to infants born to mothers in the daily supplement group. Finally, the researchers found that adherence rates were significantly higher in the twice weekly iron–folic acid supplement group compared to the once daily regime.

**What Do These Findings Mean?:**

These findings suggest that in an area of Southeast Asia with low anemia prevalence, once daily antenatal supplementation with iron–folic acid did not provide any benefits in birth weight or improved infant growth over twice weekly supplementation. Furthermore, twice weekly supplementation with iron–folic acid was associated with improved maternal adherence rates and also improved cognitive development in infants aged six months—a finding that requires further study and provides added support for the use of intermittent iron–folic acid supplementation over daily supplementation.

**Additional Information:**

Please access these websites via the online version of this summary at http://dx.doi.org/10.1371/journal.pmed.1001470.

- The World Health Organization website has comprehensive information on anemia, including a report of global estimates and the guideline Intermittent Iron and Folic Acid Supplementation in Non-Anaemic Pregnant Women
- Wikipedia provides information on iron supplementation (note: Wikipedia is a free online encyclopedia that anyone can edit; available in several languages)

## Introduction

Iron deficiency anemia is a globally important public health problem, particularly for low- and middle-income countries. Pregnant women and young children are especially vulnerable [1], with increased maternal morbidity and mortality, higher rates of preterm birth and low birth weight, and reduced infant survival, with potential long-term consequences for child growth and development [2]–[7].

Iron–folic acid (IFA) supplementation given daily from early in pregnancy has long been the recommended standard approach to prevent and treat anemia. Though efficacious, daily IFA programs have had limited success in reducing anemia prevalence in developing countries because of frequent side effects leading to poor adherence, and barriers to accessing supplements at the community level [8],[9]. In addition, the daily administration of iron to women who already have sufficient iron stores is of increasing concern because of the potential for higher ferritin levels and hemoconcentration during the antenatal period [10]–[12], with increased risk of oxidative stress and poor pregnancy outcomes [13]. High intestinal iron levels may also lead to reduced absorption of other important minerals [14].

In Viet Nam, as in most low- and middle-income countries, the current recommendation is for daily intake of antenatal IFA. However, the prevalence of anemia in pregnant women is reducing, in many areas to less than 20% [15]–[17]. In addition, IFA supplements are no longer distributed to pregnant women free of charge in Viet Nam, reducing availability at the community level. Multiple micronutrient (MMN) supplementation may also be advantageous in this setting, as micronutrient deficiencies (e.g., iodine and vitamin B12) remain prevalent in rural areas because of poor quality diet or inadequate intake [15].

In 2011, the World Health Organization (WHO) strongly recommended the use of intermittent IFA supplementation in non-anemic women in pregnancy [18]. This recommendation was based on previous studies and a Cochrane review that showed that intermittent dosing of IFA offers potential advantages for the prevention of anemia in pregnancy, including fewer side effects and increased adherence [8],[9],[19]. Absorption and retention of supplemental iron may also be more efficient when iron is administered intermittently rather than daily, as intestinal cells turn over every 5–6 d and have limited iron absorptive capacity [20],[21]. However, many of the trials supporting the recommendation [19] were limited by high risk of selection bias and significant loss to follow-up. In particular, the quality of the evidence for low birth weight, mean birth weight, premature birth, maternal anemia at term, iron deficiency anemia at term, and side effects was graded as very low.

It remains uncertain whether intermittent supplementation is advantageous in lower income settings where antenatal testing for anemia is not readily available, or whether IFA supplements that include other micronutrients would be suitable for intermittent administration. There are also minimal data on the impact of intermittent antenatal dosing on infant outcomes past the neonatal period (in particular, growth and developmental outcomes), as the majority of previous studies have followed infants only up to birth.

We conducted a community-based cluster randomised trial in a semi-rural province representative of many areas in Viet Nam to compare the effect of twice weekly provision of antenatal IFA supplementation (either alone or in combination with other micronutrients) with daily provision of IFA supplementation, on maternal and infant outcomes during the first 6 mo of life.

## Methods

### Ethical Approval

The study protocol (Text S1) was approved by the Melbourne Health Human Research Ethics Committee, and the Ha Nam Provincial Human Research Ethics Committee (Text S3). This study is registered in the Australia New Zealand Clinical Trials Registry: 12610000944033. Registration of the trial was slightly delayed. Although it was our intention to register the trial before enrolment was scheduled to commence, mitigating circumstances resulted in a delay of 30 d. This delay was because of uncertainty about the date that the supplements would be delivered to the study site by the manufacturer. Following their unexpected early arrival, a decision was made to commence the trial prior to registration, to avoid collection of survey information during the Vietnamese New Year, when many women would be visiting families in other districts. The trial has been reported according to the CONSORT guidelines for cluster randomised trials (Text S2).

### Study Design, Setting, and Participants

The trial was conducted in Ha Nam, a malaria-free province, 60 km from Hanoi, in northern Viet Nam. Ha Nam has six districts containing 116 communes, and a population of approximately 820,100, with most residents working in subsistence agriculture [22]. The average annual per capita income is US$800 [23]. To reduce treatment contamination between the different intervention groups, we used the commune as the unit of randomisation. All communes were eligible if they were located within the five rural districts of Ha Nam, and consent was given by the Ha Nam People's Committee to take part in the study. The sixth district (12 communes) included the main provincial town and was excluded because antenatal services differed from those of the rest of the province, with most deliveries taking place at the provincial hospital. All eligible pregnant women were invited to participate (90 declined because of work commitments, and 36 refused for other reasons).

Inclusion criteria were the following: residence in trial communes, age >16 y, confirmed pregnancy at <16 wk gestation, and registration with the commune health station. Women were excluded if they had a high-risk pregnancy—multi-fetal pregnancy (confirmed on palpation or ultrasound) or a significant medical condition—or if they had severe anemia (hemoglobin concentration [Hb] <80 g/l) at enrolment. Women with a significant medical condition or severe anemia were referred to the commune health station for further management.

Trained project officers recruited women, in conjunction with village and commune health station workers. At enrolment, written informed consent was obtained from all participants by the research team, with consent via thumb print for illiterate women. Demographic information was obtained using previously pilot-tested interview questions. Maternal and infant outcomes were recorded by trained commune health station staff, using structured questionnaires. Follow-up assessments of the women were undertaken at the commune health station at 32 wk gestation, birth, and 6 mo postpartum. Infants were assessed at birth and 6 mo of age.

Maternal venous blood was collected for hemoglobin and iron indices (ferritin and transferrin receptor) at enrolment, 32 wk gestation, and 6 mo postpartum. Serum folate and vitamin B12 levels were measured at enrolment and 32 wk gestation. Serum 25(OH) vitamin D concentration and urinary iodine concentration were measured at 32 wk gestation. Infant hemoglobin and ferritin were measured at 6 mo of age.

### Interventions

The trial had three intervention arms at the cluster (commune) level: (1) one tablet of IFA taken daily (60 mg elemental iron plus 0.4 mg folic acid per tablet, administered as 7 tablets/week)—this regime is the recommended approach for antenatal iron in Viet Nam, and was used as the control arm; (2) one capsule of IFA taken twice a week (60 mg elemental iron plus 1.5 mg folic acid per capsule; administered as 2 capsules/week); or (3) one capsule of MMNs taken twice a week (60 mg elemental iron plus 1.5 mg folic acid plus a variation of the dose of micronutrients in the UNIMMAP [24] daily supplement; administered as 2 capsules/week) (Table 1). Participants took supplements from enrolment until 3 mo postpartum. The IFA and MMN capsules in the twice weekly groups were identical in appearance. A placebo control was not contemplated, as it was considered unethical to withhold iron supplementation during pregnancy. Both IFA and MMN supplements were manufactured by the local Nam Ha Pharmaceutical Company (Nam Dinh, Viet Nam), which has WHO Good Manufacturing Practice certification. Tablets/capsules were independently analysed by the ALC Laboratory Group (Melbourne, Australia) to verify micronutrient dose.

**Table 1:** Composition per tablet or capsule used in the three arms of the study, in comparison to the United Nations Children's Fund/WHO UNIMAPP supplement [24].

Content	Daily IFA (Tablet)^a^	Twice Weekly IFA (Capsule)^b^	Twice Weekly MMN (Capsule)^c^	UNIMAPP Supplement
Elemental iron	60 mg	60 mg	60 mg	30 mg
Zinc			20 mg	15 mg
Iodine			300 µg	150 µg
Copper			4 mg	2 mg
Selenium			130 µg	65 mg
Vitamin A			1.6 mg	0.8 mg
Thiamine			2.8 mg	1.4 mg
Riboflavin			2.8 mg	1.4 mg
Niacin			36 mg	18 mg
Vitamin B6			3.8 mg	1.9 mg
Vitamin B12			5.2 µg	2.6 µg
Folic acid	0.4 mg	1.5 mg	1.5 mg	0.4 mg
Vitamin C			140 mg	70 mg
Vitamin D			400 IU	200 IU
Vitamin E			20 mg	10 mg

a Seven tablets given each week = 420 mg elemental iron, 2.8 mg folic acid/week.

b Two capsules given each week = 120 mg elemental iron, 3 mg folic acid/week.

c Two capsules given each week = 120 mg elemental iron, 3 mg folic acid, 40 mg zinc, and approximately four times the recommended daily intake of other micronutrients/week.

Each woman was visited every 6 wk at home, to distribute the intervention with written instructions for the next 6 wk, and collect information on adherence, side effects, and pregnancy complications. Intake was not supervised.

The research team did not provide medical treatment for women in the trial. However, the field teams worked closely with commune health workers, and women with hemoglobin levels less than 80 g/l, clinical depression, or other significant illness were referred to the commune health station for further management. Following completion of the trial, feedback was given to women about ferritin, iodine, and hemoglobin results, along with nutrition information.

### Objectives

The primary objective was to compare the effect of twice weekly antenatal provision of (1) IFA supplementation or (2) MMN supplementation with daily provision of antenatal IFA supplementation, on maternal and infant outcomes.

### Outcomes

The primary outcome was infant birth weight. Secondary outcomes were maternal hemoglobin and ferritin at 32 wk, and infant length-for-age *z*-scores, hemoglobin, ferritin, and cognitive development at 6 mo of age. All interventions were delivered at the level of the commune, and outcomes were measured at the individual level as follows.

#### Birth outcome measures

Gestational age at birth was calculated from estimated gestational age recorded by trans-abdominal ultrasound performed at the district hospital if available (1,041 infants), and if not, according to the date of last menstruation (136 infants). Stillbirths (fetal deaths at or after 28 wk of pregnancy) and early neonatal deaths (defined here as deaths within the first 24 h after birth) were documented by trained commune health station midwives.

#### Anthropometric measures

Research staff recorded triplicate measurements of anthropometric measures, a second observer checked all measurements, and the median measurement was used for analysis. Digital infant weighing scales with precision to the nearest 10 g (BF20510, Laica) were provided for birth weight measurements in each commune health station and district hospital. For home deliveries, commune and village health workers assessed the woman and baby within 72 h of birth. Maternal height, birth crown–heel length, and infant crown–heel length were measured using a portable Shorr Board (Shorr Productions). Head circumference was measured using a non-stretchable measuring tape. Maternal weight and infant weight at 6 mo were measured using electronic Seca 876 or 890 scales, with precision to the nearest 100 g. Growth (length for age, weight for age, and weight for length) was evaluated using the 2006 WHO Child Growth Standards [25].

Definitions used were: low birth weight, <2,500 g; very low birth weight, <1,500 g; and preterm birth, live birth before 37 wk gestation. Anthropometric *z*-scores were calculated using WHO Anthro (version 3.2.2, January 2011) [26]. Being stunted, wasted, or underweight was defined as having a *z*-score less than two standard deviations (SDs) below WHO growth standards on length for age, weight for length, or weight for age, respectively [25].

#### Laboratory measurements

Maternal and infant hemoglobin were measured using HemoCue, a portable photometer (HemoCue Hemoglobin Systems). Five millilitres of maternal venous blood was collected for iron indices (ferritin and transferrin receptor) and serum folate, vitamin B12, and vitamin D levels; maternal urine was collected for urinary iodine concentration at 32 wk gestation; and 1 ml of infant venous blood was collected for serum ferritin. Samples were frozen at −20°C and transported to the Alfred Hospital, Melbourne, for testing. Serum B12, folate, and ferritin were analysed using a chemiluminescent microparticle assay, and soluble transferrin receptor using an immunoturbimetric assay (Architect ci16200; Abbott Diagnostics). A Waters TQD mass spectrometer was used to quantify 25(OH) vitamin D concentration. Urinary samples were frozen at –20°C, and transported to the laboratory of the National Hospital of Endocrinology in Hanoi. Urinary iodine concentration was determined by means of the Sandell-Kolthoff reaction, as recommended by WHO, the United Nations Children's Fund, and the International Council for the Control of Iodine Deficiency Disorders [27]. Definitions of deficiency for maternal samples were as follows: anemia, Hb<110 g/l; iron deficiency, serum ferritin <15 µg/l; vitamin B12 deficiency, serum B12<150 pmol/l; folate deficiency, serum folate <10 nmol/l [2],[28]; iron deficiency anemia, Hb<110 g/l and ferritin <15 µg/l [2]; high ferritin, ferritin >41 µg/l [13]; hemoconcentration, Hb>130 g/l [12]; iodine deficiency, urinary iodine <150 µg/l [27]; and vitamin D insufficiency, serum 25(OH) vitamin D<75 nmol/l [29]. Definitions of deficiency for infant samples were as follows: anemia, hemoglobin <110 g/l; iron deficiency, serum ferritin <9 µg/l [30]; and iron deficiency anemia, hemoglobin <110 g/l plus serum ferritin <9 µg/l. Transferrin index was calculated using the ratio of transferrin receptor to log (base 10) serum ferritin.

#### Developmental outcome measures

Measurement of infant development was performed at 6 mo of age using the *Bayley Scales of Infant and Toddler Development, 3rd edition* (BSID III) [31]. The BSID III scales were translated from English into Vietnamese and back-translated to English for verification. BSID III administrators were community-based psychologists experienced in early child development assessment, and were trained by a local Vietnamese expert in BSID III following the guidelines of the BSID III administration manual [31]. The BSID III was used to conduct direct infant developmental assessments for cognitive, language, and motor domains, and rating of mothers determined the socio-emotional and adaptive behaviour scores.

The BSID III cognitive scale is composed of 91 items that assess sensorimotor development, exploration and manipulation, object relatedness, concept formation, and memory. Each test subscale gave a total raw score based on the number of items passed, which was then converted to a scaled score. The five subscales were then converted to composite scores using normative test data, based on the guidelines of the BSID III manual, and these composite scores were used in the final analysis. Each composite score has a normative mean value of 100 and a SD of 15 quotient points. The cognitive composite score was based on the cognitive scaled score, the language composite score was a combination of the receptive and expressive scaled scores, and the motor composite score was a combination of the fine and gross motor scaled scores. The instrument was previously pilot-tested in Ha Nam Province and has been adapted to the local cultural context. Mild developmental delay was defined as a composite score of one to two SDs below the mean, moderate delay as more than two to three SDs below the mean, and severe delay as more than three SDs below the mean.

### Adherence

Adherence was calculated as the total number of supplements consumed by each woman, divided by the total number of supplements expected to be consumed by each woman. The number of supplements consumed was determined from the number of tablets taken as reported by the women, and recorded by the monitors during the visits every 6 wk.

### Sample Size

Observational studies indicated that on average there were ten eligible pregnant women of <16 wk gestation per commune (mean 10, range 2–20) [32]. It was expected that 5% of women would be excluded because of pregnancy complications, and 15% would be lost to follow-up. Based on this, and using a superiority trial design, sample size calculations indicated that a minimum of 34 communes (408 pregnant women) would be needed per treatment arm, i.e., a total of 1,224 women from 102 communes, to detect differences of 6 g/l for hemoglobin (power = 98%, SD = 12 g/l, intra-cluster correlation coefficient [ICC] = 0.12), 100 g for birth weight (power = 81%, SD = 389 g, ICC = 0.03), and 0.3 for length-for-age *z*-score (power = 93%, SD = 0.9, ICC = 0.05) with 80% statistical power and a significance level of 0.05. The ICC was estimated from previously published literature [33]–[35].

### Randomisation and Masking

Allocation was based on communes, and all communes in the province, other than those in the principal town district, were randomly assigned to one of three treatment groups. Randomisation was performed by an independent statistician not involved in the study and blinded to the identity of the communes, using ‘ralloc’ in Stata (StataCorp). Supplements were received from the manufacturing company in blister packs, with a code A, B, or C embossed on each blister pack. The intermittent IFA and MMN capsules were identical in both appearance and packaging. The manufacturing company confidentially notified the chairperson of the Data Monitoring and Safety Committee at the University of Melbourne of the allocation code, and the code was kept in a locked file cabinet at the University of Melbourne, Australia. The investigators, field staff, and participants were blinded to the codes of the intermittent supplement groups throughout the study and during data analysis. Laboratory staff were unaware of the intervention groups. It was not possible to blind the field team to the daily supplementation arm, but participants were not informed about the dosing frequency of the intervention being given in other communes. The allocation code was broken at the completion of data analysis. An independent team undertook the BSID III assessments, and were blinded to the intervention arms.

### Statistical Analysis

Data were double entered into a customised database by a trained data entry officer in the province, and checked and cleaned by an epidemiologist in Hanoi. Data were analysed according to the group to which women were randomised, using modified intention to treat because of loss to follow-up. No imputation of the missing data was performed for those lost to follow-up. We examined baseline characteristics of individual pregnant women across treatment groups to assess randomisation. Estimates (95% confidence intervals [CIs]) of the mean difference (MD) in continuous outcome measures between the daily and weekly trial arms were calculated using linear regression, and logistic regression was used for binary outcomes. For hemoglobin and ferritin, linear regression models included the baseline measurement as a covariate. Since ferritin and transferrin receptor levels were positively skewed, they were log_e_ transformed before analysis, and comparison between trial arms was presented as the ratio of geometric means (GMs). Robust standard errors were calculated using the Huber-White Sandwich estimator to account for study design (i.e., clustering at commune level). The ICC was calculated for continuous and binary outcome measures using one-way analysis of variance with commune as the group variable. All statistical analyses were performed using Stata, version 11.2 (StataCorp).

## Results

**Figure 1:**
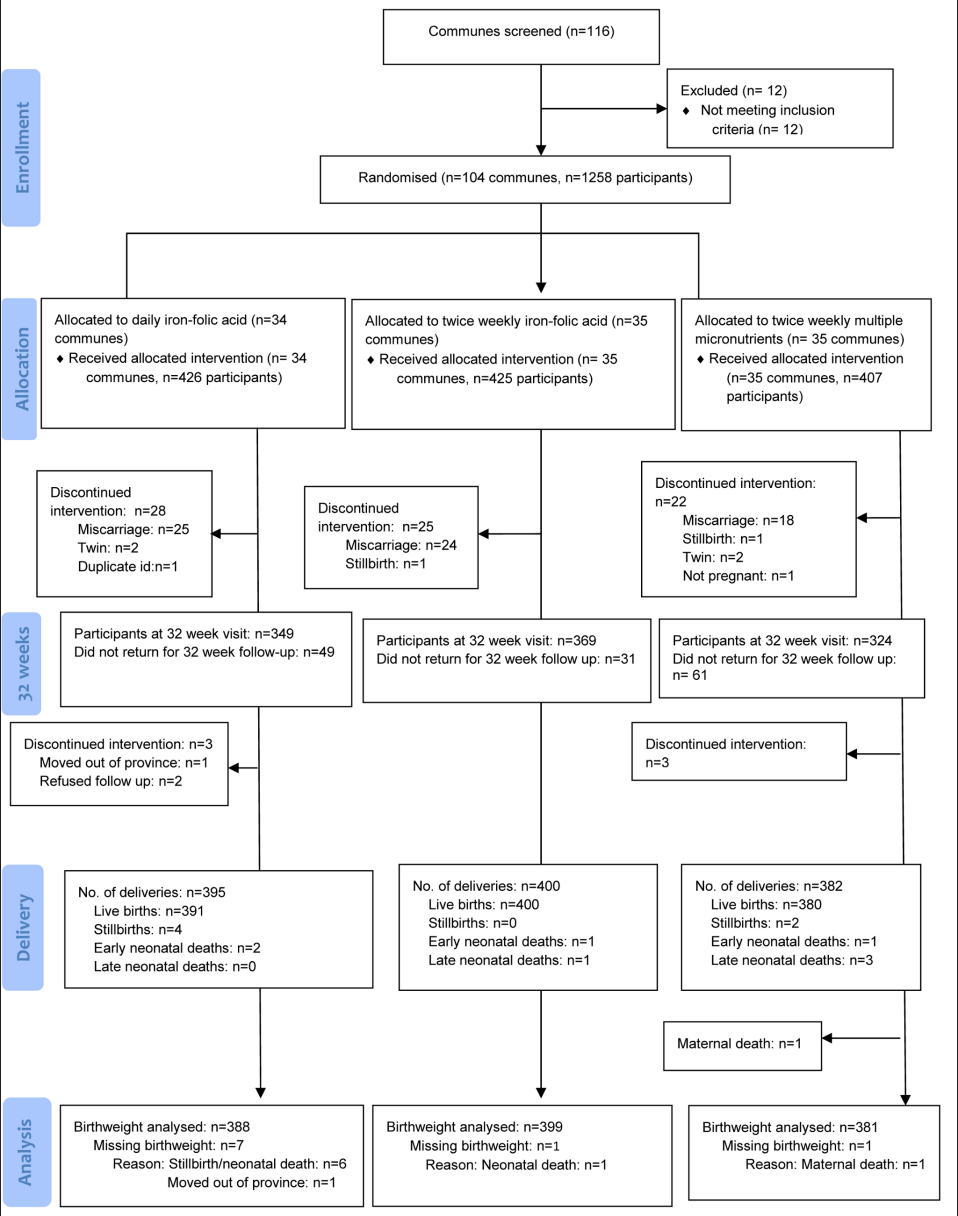
Trial profile. Late neonatal death was defined as death after the first 24 h and before 24 d of life.

Enrolment occurred between 28 September 2010 and 5 November 2010. A total of 1,258 pregnant women from the 104 eligible communes were enrolled. The distribution of each baseline characteristic is presented in Table 2. The trial profile is presented in Figure 1. Loss to follow-up was 8.9% (38/426) in the daily IFA group, 6.1% (26/425) in the twice weekly IFA group, and 6.4% (26/407) in the twice weekly MMN group, for the primary outcome of birth weight. Baseline maternal characteristics of mothers of infants who had birth weight measured, were similar to those who were lost to follow-up, within each treatment arm (Table S1).

**Table 2:** Baseline characteristics of women at enrolment.

Characteristic	Daily IFA	Twice Weekly IFA	Twice Weekly MMN
**Number of communes**	34	35	35
**Number enrolled**	426	425	407
**Maternal age (years)**			
**Mean age**	26.2 [5.1]	27.3 [4.8]	26.9 [5.0]
<20 y	24 (5.6)	8 (1.9)	18 (4.4)
20–24 y	152 (35.8)	129 (30.3)	119 (29.2)
25–29 y	160 (37.7)	170 (40.0)	167 (41.0)
30–34 y	54 (12.7)	87 (20.4)	65 (16.0)
≥35 y	35 (8.2)	32 (7.5)	38 (9.3)
**Maternal education**			
Primary school (up to year 5)	78 (18.3)	54 (12.7)	66 (16.2)
Secondary school (years 6–9)	199 (46.8)	230 (54.0)	199 (48.9)
High school (years 10–12)	105 (24.6)	94 (22.1)	108 (26.5)
Post-secondary qualification	43 (10.1)	48 (11.3)	34 (8.4)
**Employment**			
Farmer	170 (40.0)	181 (42.5)	162 (39.8)
Factory worker	69 (16.2)	60 (14.1)	53 (13.0)
Trader	75 (17.7)	85 (20.0)	77 (18.9)
Government official	44 (10.4)	55 (12.9)	41 (10.1)
Clerk	17 (4.0)	11 (2.6)	18 (4.4)
Not in income-generating work	46 (10.8)	27 (6.3)	49 (12.0)
Other	4 (0.9)	7 (1.6)	7 (1.7)
**Mean gestational age (weeks)**	12.40 [3.3]	12.06 [3.3]	12.15 [3.5]
**Gestation at ≤12 wk**	195 (45.8)	215 (50.6)	196 (48.2)
**Gravidity**			
0	149 (35.1)	107 (25.1)	130 (31.9)
1	139 (32.7)	155 (36.4)	156 (38.3)
2–3	117 (27.5)	140 (32.9)	106 (26.0)
4–5	19 (4.5)	22 (5.2)	13 (3.2)
≥6	1 (0.2)	2 (0.5)	1 (0.2)
**Previous stillbirth or early neonatal death**	13 (4.7)	25 (7.9)	17 (6.9)
**Mean maternal weight (kg)**	47.2 [5.5]	47.0 [5.3]	46.5 [5.5]
**Mean maternal height (cm)** a	154.0 [4.8]	154.0 [4.8]	153.1 [4.7]
**Mean mid-upper-arm circumference (cm)**	24.1 [4.2]	23.8 [1.9]	23.8 [2.2]
**Mean body mass index (kg/m^2^)** a	19.9 [1.9]	19.9 [1.9]	19.9 [2.1]

Values are mean [SD], or number (percent).

a Data on height missing for one woman.

### Birth Outcomes

#### Primary outcome

Mean birth weight for all newborns was 3,148 g (SD 416) (Table 3). The distribution of birth weight was similar in infants born to women who took twice weekly IFA compared to those who took daily IFA (MD 28 g; 95% CI −22 to 78), and in infants born to mothers who took twice weekly MMN compared to those who took daily IFA (MD −36.8 g; 95% CI −82 to 8.2) (Table 3). Maternal and neonatal outcomes, with and without adjustment for potential confounders, including maternal age, parity, infant gender, and gestational age, are presented in Table 4.

**Table 3:** Birth outcomes, with mean difference or odds ratio, for comparison of the intervention groups.

Birth Outcome	Number (Percent) of Infants	Mean [SD]	MD or Odds Ratio (95% CI)^a^	*p*-Value	ICC
**Birth weight (g)** b					0.00
Daily IFA	388/395 (98.2)	3,151 [419]	Reference		
Twice weekly IFA	399/400 (99.8)	3,178 [376]	28.0 (−22.1 to 78.1)	0.27	
Twice weekly MMN	381/382 (99.7)	3,114 [450]	−36.8 (−81.9 to 8.2)	0.11	
**Low birth weight (<2,500 g)** b					0.00
Daily IFA	17/388 (4.4)	N/A	Reference		
Twice weekly IFA	9/399 (2.3)	N/A	0.50 (0.22 to 1.14)	0.10	
Twice weekly MMN	16/381(4.2)	N/A	0.96 (0.49 to 1.87)	0.90	
**Very low birth weight (<1,500 g)** b					0.00
Daily IFA	2/388 (0.5)	N/A	Reference		
Twice weekly IFA	0/399 (0)	N/A	N/A	N/A	
Twice weekly MMN	3/381 (0.8)	N/A	1.53 (0.23 to 10.1)	0.66	
**Gestation at birth (weeks)** c					0.007
Daily IFA	395/395 (100)	38.7 [2.85]	Reference		
Twice weekly IFA	400/400 (100)	38.6 [2.52]	−0.11 (−0.52 to 0.30)	0.60	
Twice weekly MMN	381/382 (99.7)	38.6 [2.78]	−0.12 (−0.53 to 0.30)	0.57	
**Preterm (<37 wk)** c					0.00
Daily IFA	64/395 (16.2)	N/A	Reference		
Twice weekly IFA	62/400 (15.5)	N/A	0.95 (0.66 to 1.38)	0.79	
Twice weekly MMN	72/381 (18.9)	N/A	1.21 (0.85 to 1.72)	0.29	
**Stillbirth**					0.00
Daily IFA	4/395 (1.0)	N/A	Reference		
Twice weekly IFA	0/400 (0)	N/A	N/A	N/A	
Twice weekly MMN	2/382 (0.5)	N/A	0.51 (0.11 to 2.60)	0.42	
**Early neonatal death**					0.00
Daily IFA	2/395 (0.51)	N/A	Reference		
Twice weekly IFA	1/400 (0.25)	N/A	0.49 (0.05 to 5.00)	0.55	
Twice weekly MMN	1/382 (0.26)	N/A	0.52 (0.05 to 5.05)	0.57	
**Birth length (cm)** d					0.29
Daily IFA	197/395 (49.9)	49.3 [3.74]	Reference		
Twice weekly IFA	182/400 (45.5)	49.4 [2.03]	0.16 (−0.98 to 1.30)	0.78	
Twice weekly MMN	169/382 (44.2)	49.0 [2.78]	−0.24 (−1.48 to 1.01)	0.71	
**Head circumference (cm)** e					0.17
Daily IFA	199/395 (50.4)	32.9 [1.96]	Reference		
Twice weekly IFA	185/400 (46.3)	32.2 [1.99]	−0.70 (−1.26 to −0.14)	0.02	
Twice weekly MMN	169/382 (44.2)	33.1 [2.39]	0.23 (−0.38 to 0.84)	0.46	

a Model adjusted for cluster randomisation.

b Data missing on nine infants (seven in daily IFA, one in twice weekly IFA, one in twice weekly MMN).

c Data missing on one infant in twice weekly MMN group.

d Data missing on 629 infants (198 in daily IFA, 218 in twice weekly IFA, 213 in twice weekly MMN).

e Data missing on 624 infants (196 in daily IFA, 215 in twice weekly IFA, 213 in twice weekly MMN).

N/A, not applicable.

**Table 4:** Maternal and neonatal outcomes, with mean difference or odds ratio, for comparison of the intervention groups, with and without adjustment for potential confounders.

Maternal and Neonatal Outcomes	Model Adjusted for Cluster Randomisation Only		Model Adjusted for Other Potential Confounders^a^	
	MD/Odds Ratio/GM Ratio (95%)^a^	*p*-Value	MD/Odds Ratio/GM Ratio (95% CI)^b^	*p*-Value
**Birth weight (g)**				
Daily IFA	Reference		Reference	
Twice weekly IFA	28.0 (−22.1 to 78.1)	0.27	32.5 (−16.3 to 81.3)	0.19
Twice weekly MMN	−36.8 (−81.9 to 8.2)	0.11	−19.7 (−69.7 to 30.3)	0.44
**Hemoglobin(g/l) at 32 wk** b				
Daily IFA	Reference		Reference	
Twice weekly IFA	0.02 (−2.06 to 2.10)	0.99	0.03 (−2.04 to 2.09)	0.98
Twice weekly MMN	−1.02 (−3.55 to 1.52)	0.43	−1.07 (−2.04 to 2.10)	0.40
**Ferritin (µg/l) at 32 wk** b				
Daily IFA	Reference		Reference	
Twice weekly IFA	0.73 (0.67 to 0.80)	<0.001	0.72 (0.67 to 0.79)	<0.001
Twice weekly MMN	0.62 (0.57 to 0.68)	<0.001	0.61 (0.56 to 0.67)	<0.001
**Infant length-for-age ** ***z*** **-score**				
Daily IFA	Reference		Reference	
Twice weekly IFA	−0.13 (−0.28 to 0.02)	0.09	−0.14 (−0.29 to 0.01)	0.07
Twice weekly MMN	−0.04 (−0.20 to 0.12)	0.62	−0.05 (−0.20 to 0.11)	0.55
**Infant hemoglobin (g/l)**				
Daily IFA	Reference		Reference	
Twice weekly IFA	−0.55 (−2.44 to 1.33)	0.56	−0.58 (−2.52 to 1.35)	0.55
Twice weekly MMN	0.75 (−1.26 to 2.76)	0.46	0.74 (−1.31 to 2.78)	0.48
**Infant cognitive development**				
Daily IFA	Reference		Reference	
Twice weekly IFA	1.91 (0.25 to 3.56)	0.02	1.89 (0.23 to 3.56)	0.03
Twice weekly MMN	0.78 (−0.76 to 2.33)	0.32	0.79 (−0.74 to 2.32)	0.31

a Model has been adjusted for maternal age, parity, and cluster randomisation. In addition to these variables, birth weight has also been adjusted for infant gender and gestational age.

b Baseline values included in model.

#### Secondary outcomes

Head circumference was measured in 553/1,177 infants (47%). Head circumference was significantly smaller in the twice weekly IFA group compared to the daily IFA group (MD −0.70, 95% CI −1.26 to −0.14). No difference was seen in infants born to women who received twice weekly MMN compared to daily IFA (MD 0.23; 95% CI −0.38 to 0.84). No clinically significant differences in gestational age, risk of prematurity, stillbirth, or early neonatal death were seen between the different supplement groups (Table 3). Congenital anomalies were seen in 0.2% of children (2/1,177).

### Maternal Hemoglobin and Micronutrient Outcomes

At enrolment, mean hemoglobin was 123 g/l (SD 12.3), and 159/1,258 (12.6%) women were anemic. At 32 wk gestation, 110/1,023 (10.8%) were anemic. No difference in hemoglobin was found between the different supplement groups at 32 wk gestation, taking into account baseline hemoglobin (Table 5).

**Table 5:** Laboratory values at 32 wk gestation, with mean difference, geometric mean ratio, or odds ratio, for comparison of the intervention groups.

Laboratory Test	Number (Percent)	Mean [SD]/GM (95% CI)	MD, Odds Ratio, or GM Ratio (95% CI)^a^	*p*-Value	ICC
**Hemoglobin (g/l)** b **^,^** c					0.03
Daily IFA	344/350 (98.3)	124.9 [12.2]	Reference		
Twice weekly IFA	361/368 (98.1)	123.8 [11.7]	0.02 (−2.06 to 2.10)	0.99	
Twice weekly MMN	318/324 (98.1)	123.4 [12.4]	−1.02 (−3.55 to 1.52)	0.43	
**Anemia (Hb<110 g/l)** b **^,^** c					0.03
Daily IFA	35/344 (10.2)	N/A	Reference		
Twice weekly IFA	33/361 (9.1)	N/A	0.81 (0.45 to 1.44)	0.46	
Twice weekly MMN	42/318 (13.2)	N/A	1.35 (0.72 to 2.52)	0.36	
**Ferritin (µg/l)** b **^,^** d					0.08
Daily IFA	342/350 (97.7)	34.5 (32.1 to 37.1)	Reference		
Twice weekly IFA	361/368 (98.1)	25.7 (24.2 to 27.2)	0.73 (0.67 to 0.80)	<0.001	
Twice weekly MMN	316/324 (97.5)	21.7 (20.2 to 23.3)	0.62 (0.57 to 0.68)	<0.001	
**Iron deficiency (ferritin <15 µg/l)** b **^,^** d					0.04
Daily IFA	40/342 (11.7)	N/A	Reference		
Twice weekly IFA	59/361 (16.3)	N/A	1.52 (0.94 to 2.45)	0.09	
Twice weekly MMN	90/316 (28.5)	N/A	3.11 (1.98 to 4.91)	<0.001	
**Transferrin receptor** d					0.07
Daily IFA	342/350 (97.7)	3.22 (3.14 to 3.31)	Reference		
Twice weekly IFA	361/368 (98.1)	3.54 (3.26 to 3.63)	1.10 (1.05 to 1.15)	<0.001	
Twice weekly MMN	316/324 (97.5)	3.58 (3.47 to 3.68)	1.11 (1.06 to 1.16)	<0.001	
**Transferrin index** e					0.09
Daily IFA	340/350 (97.1)	2.14 (2.06 to 2.23)	Reference		
Twice weekly IFA	358/368 (97.3)	2.55 (2.46 to 2.65)	1.19 (1.12 to 1.27)	<0.001	
Twice weekly MMN	312/324 (96.3)	2.75 (2.63 to 2.88)	1.29 (1.20 to 1.38)	<0.001	
**Iron deficiency anemia (Hb<110 g/l, ferritin <15 µg/l)** c					0.03
Daily IFA	3/342 (0.9)	N/A	Reference		
Twice weekly IFA	8/361 (2.2)	N/A	2.55 (0.72 to 9.10)	0.15	
Twice weekly MMN	19/315 (6.0)	N/A	7.23 (2.21 to 23.62)	<0.001	
**High ferritin (>41 µg/l)**					0.05
Daily IFA	207/342 (60.5)	N/A	Reference		
Twice weekly IFA	149/361 (41.3)	N/A	0.45 (0.33 to 0.62)	<0.001	
Twice weekly MMN	99/316 (31.3)	N/A	0.29 (0.21 to 0.42)	<0.001	
**Hemoconcentration (Hb>130 g/l)**					0.04
Daily IFA	112/344 (32.6)	N/A	Reference		
Twice weekly IFA	97/361 (26.9)	N/A	0.76 (0.52 to 1.11)	0.15	
Twice weekly MMN	87/318 (27.4)	N/A	0.78 (0.50 to 1.21)	0.27	
**Folate (nmol/l)**					0.04
Daily IFA	342/350 (97.7)	28.9 [10.7]	Reference		
Twice weekly IFA	361/368 (98.1)	29.2 [9.9]	0.28 (−1.25 to 1.81)	0.72	
Twice weekly MMN	315/324 (97.2)	24.9 [8.8]	−4.04 (−5.56 to −2.53)	<0.001	
**B12 (pmol/l)**					
Daily IFA	342/350 (97.7)	239.4 [78.1]	Reference		
Twice weekly IFA	361/368 (98.1)	243.9 [83.6]	4.11 (−10.45 to 18.7)	0.58	
Twice weekly MMN	316/324 (97.5)	249.2 [90.3]	9.44 (−4.74 to 23.6)	0.19	
**Iodine (µg/l)**					0.11
Daily IFA	348/350 (99.4)	44.8 (40.8 to 49.2)	Reference		
Twice weekly IFA	368/368 (100)	50.2 (46.7 to 54.1)	1.12 (0.91 to 1.37)	0.27	
Twice weekly MMN	322/324 (99.4)	53.3 (48.7 to 58.9)	1.19 (0.96 to 1.47)	0.10	
**Vitamin D (mmol/l)**					0.07
Daily IFA	327/350 (93.4)	67.8 [20.6]	Reference		
Twice weekly IFA	340/368 (92.4)	68.0 [21.5]	0.15 (−3.69 to 4.0)	0.94	
Twice weekly MMN	293/324 (90.4)	76.3 [23.8]	8.44 (3.71 to 13.2)	0.001	

a Model adjusted for cluster randomisation.

b Adjusted for baseline values.

c Data missing on 19 women (six in daily IFA arm, seven in twice weekly IFA arm, six in twice weekly MMN arm).

d Data missing on 19 women (four in daily IFA arm, seven in twice weekly IFA arm, eight in twice weekly MMN arm).

e Data missing on 32 women (ten in daily IFA arm, ten in twice weekly IFA arm, 12 in twice weekly MMN arm).

N/A, not applicable.

GM ferritin was 75.6 µg/l (95% CI 72.7 to 78.6) at enrolment, with 2.2% (95% CI 1.3 to 3.0) of women iron deficient. Ferritin decreased from enrolment to 32 wk in all groups (*p*<0.001), with 189/1,019 (18.6%) women iron deficient at 32 wk. The prevalence of iron deficiency at 32 wk gestation was 11.7% in the daily IFA group, 16.3% in the twice weekly IFA group, and 28.5% in the MMN group. Women in the twice weekly IFA group had lower ferritin levels at 32 wk than those in the daily IFA group (GM ratio 0.73, 95% CI 0.67 to 0.80). Lower ferritin levels were also found in women who took twice weekly MMN compared to those who took daily IFA (GM ratio 0.62, 95% CI 0.57 to 0.68).

At 32 wk gestation, the prevalence of other micronutrient deficiencies was 92.5% for iodine, 8.5% for B12, 2.4% for folate, and 60.6% for vitamin D insufficiency (Table 5).

### Infant Outcomes at 6 mo of Age

#### Hemoglobin and iron

Mean hemoglobin at 6 mo of age was 110.4 g/l (SD 11.3), and 516/1,024 (50%) infants were anemic. The values for mean hemoglobin and prevalence of anemia in infants at 6 mo of age were similar in infants born to women taking twice weekly IFA or MMN supplements compared to those taking daily IFA during pregnancy. GM ferritin was 28.4 µg/l (95% CI 26.8 to 30.1), and 8.9% of infants were iron deficient (Table 6).

**Table 6:** Infant laboratory values at 6 mo, with mean difference, geometric mean ratio, or odds ratio, for comparison of the intervention groups.

Laboratory Test	Number (Percent) of Infants	Mean [SD]/GM (95% CI)	MD, Odds Ratio, or GM Ratio (95% CI)^a^	*p*-Value	ICC
**Hemoglobin (g/l)** b					0.03
Daily IFA	335/351 (95.4)	110.3 [11.4]	Reference		
Twice weekly IFA	359/363 (98.9)	109.8 [11.1]	−0.55 (−2.44 to 1.33)	0.56	
Twice weekly MMN	330/335 (97.9)	111.1 [11.6]	0.75 (−1.26 to 2.76)	0.46	
**Anemia** b					0.02
Daily IFA	158/335 (47.2)	N/A	Reference		
Twice weekly IFA	192/359 (53.5)	N/A	1.29 (0.95 to 1.75)	0.11	
Twice weekly MMN	166/330 (50.3)	N/A	1.13 (0.82 to 1.57)	0.45	
**Ferritin (µg/l)** c					0.00
Daily IFA	306/351 (87.2)	29.5 (26.6 to 32.8)	Reference		
Twice weekly IFA	325/363 (89.5)	27.0 (24.5 to 29.7)	0.91 (0.80 to 1.05)	0.20	
Twice weekly MMN	298/335 (88.9)	28.8 (26.1 to 31.9)	0.98 (0.83 to 1.14)	0.76	
**Iron deficiency (ferritin <9 µg/l)** c					0.00
Daily IFA	33/306 (10.8)	N/A	Reference		
Twice weekly IFA	27/325 (8.3)	N/A	0.75 (0.44 to 1.29)	0.30	
Twice weekly MMN	23/298 (7.7)	N/A	0.60 (0.42 to 1.15)	0.16	
**Iron deficiency anemia (ferritin <9 µg/l, Hb<110 g/l)** d					0.00
Daily IFA	20/306 (6.5)	N/A	Reference		
Twice weekly IFA	23/325 (7.1)	N/A	1.09 (0.58 to 2.06)	0.79	
Twice weekly MMN	18/297 (6.1)	N/A	0.92 (0.53 to 1.61)	0.78	

a Model adjusted for cluster randomisation.

b Data missing for 25 infants (16 in daily IFA group, four in twice weekly IFA group, five in twice weekly MMN group).

c Data missing for 120 infants (45 in daily IFA group, 38 in twice weekly IFA group, 37 in twice weekly MMN group).

d Data missing for 121 infants (45 in daily IFA group, 38 in twice weekly IFA group, 38 in twice weekly MMN group).

N/A, not applicable.

#### Growth

Prevalence of stunting at 6 mo of age was 6.4% (67/1,046). Infant growth outcomes, with adjustment for potential confounders, are presented in Table 7. There was no difference in infant length-for-age *z*-scores at 6 mo of age in the twice weekly IFA group compared to the daily IFA group (MD −0.14, 95% CI −0.29 to 0.02), nor in the twice weekly MMN group compared to the daily IFA group (MD −0.04, 95% CI −0.20 to 0.11).

**Table 7:** Infant growth and developmental outcomes at 6 mo of age, with mean difference or odds ratio, for comparison of the intervention groups, adjusted for potential confounders.

Category	Outcome	Number (Percent) of Infants	Mean [SD]	MD or Odds Ratio (95% CI)^a^	*p*-Value	ICC
**Infant growth outcomes at 6 mo**	**Length** b					0.006
	Daily IFA	350/351 (99.7)	66.1 [2.3]	Reference		
	Twice weekly IFA	363/363 (100)	65.8 [2.1]	−0.33 (−0.70 to 0.03)	0.08	
	Twice weekly MMN	333/335 (99.4)	66.0 [2.4]	−0.13 (−0.49 to 0.23)	0.47	
	**Length-for-age ** ***z*** **-score** b					
	Daily IFA	350/351 (99.7)	−0.53 [0.93]	Reference		0.005
	Twice weekly IFA	363/363 (100.0)	−0.67 [0.91]	−0.14 (−0.29 to 0.02)	0.08	
	Twice weekly MMN	333/335 (99.4)	−0.57 [0.98]	−0.04 (−0.20 to 0.11)	0.61	
	**Stunting** b					0.02
	Daily IFA	23/350 (6.6)	N/A	Reference		
	Twice weekly IFA	22/363 (6.1)	N/A	0.95 (0.49 to 1.83)	0.88	
	Twice weekly MMN	23/333 (6.6)	N/A	1.03 (0.54 to 1.97)	0.94	
**Infant developmental outcomes at 6 mo**	**Cognitive** c					0.00
	Daily IFA	350/351 (99.7)	100.2 [11.4]	Reference		
	Twice weekly IFA	363/363 (100)	102.1 [10.9]	1.89 (0.23 to 3.56)	0.03	
	Twice weekly MMN	335/335 (100)	101.2 [9.9]	0.79 (−0.74 to 2.32)	0.31	
	**Language** c					
	Daily IFA	350/351 (99.7)	94.0 [8.0]	Reference		0.01
	Twice weekly IFA	363/363 (100)	94.8 [8.2]	0.89 (−0.44 to 2.22)	0.19	
	Twice weekly MMN	335/335 (100)	94.8 [7.7]	0.83 (−0.54 to 2.20)	0.23	
	**Motor** c					
	Daily IFA	350/351 (99.7)	112.3 [18.2]	Reference		0.0006
	Twice weekly IFA	363/363 (100)	113.4 [19.0]	0.99 (−1.59 to 3.57)	0.45	
	Twice weekly MMN	335/335 (100)	112.5 [18.4]	0.12 (−2.98 to 3.21)	0.94	
	**Socio-emotional** c					
	Daily IFA	350/351 (99.7)	81.2 [10.5]	Reference		0.03
	Twice weekly IFA	363/363 (100)	81.9 [11.9]	0.79 (−1.32 to 2.91)	0.46	
	Twice weekly MMN	335/335 (100)	82.0 [11.5]	0.85 (−0.74 to 2.43)	0.29	
	**Adaptive behaviour** d					
	Daily IFA	351/351 (100)	97.2 [9.9]	Reference		0.02
	Twice weekly IFA	362/363 (99.7)	97.4 [9.6]	0.15 (−1.62 to 1.92)	0.87	
	Twice weekly MMN	334/335 (99.7)	97.7 [10.1]	0.48 (−0.90 to 1.87)	0.49	

a Model adjusted for maternal age, parity, and cluster randomisation.

b Data missing for three infants (one in daily IFA group, two in twice weekly MMN group).

c Data missing for one infant (one in daily IFA group).

d Data missing for two infants (one in twice weekly IFA group, two in twice weekly MMN group).

N/A, not applicable.

#### Development

Mean cognitive composite scores at 6 mo of age were within the normal range expected from BSID III normative data. Mean scores were 100.2 (SD 11.4) in the daily IFA group, 102.1 (SD 10.9) in the twice weekly IFA group, and 101.2 (SD 9.9) in the twice weekly MMN group (Table 7). At 6 mo of age, 15.7% of infants had mild cognitive delay (cognitive composite score 1 to 2 SDs below the mean), 1.4% had moderate cognitive delay (cognitive composite score >2 to 3 SDs below the mean), and 0.95% had severe cognitive delay (>3 SDs below the mean). Infant developmental outcomes, with adjustment for potential confounders, are presented in Table 7. Infants whose mothers received twice weekly IFA had higher cognitive composite scores compared to those who received daily IFA (MD 1.89, 95% CI 0.23 to 3.56). There was no difference in cognitive scores in infants born to mothers who received twice weekly MMN compared to those who received daily IFA (MD 0.79, 95% CI −0.74 to 2.32). No difference in other developmental composite scores (language, motor, socio-emotional, or adaptive behavior) was found between the twice weekly supplement groups compared to the daily IFA group.

### Adherence and Side Effects

The main reported side effects were nausea 28.7% (143/1,255), vomiting 11.4% (143/1,255), and constipation 27.3% (343/1,255). There was no significant difference in nausea or vomiting between the twice weekly IFA and daily IFA groups (*p*>0.05). Prevalence of nausea and vomiting was significantly higher in the twice weekly MMN group compared to the daily IFA group. The median level of adherence was 91% in the daily IFA group, 96% in the twice weekly IFA group, and 85% in the twice weekly MMN group. Adherence was significantly higher in the twice weekly IFA group compared to the daily IFA group (*p* = 0.01), and significantly lower in the twice weekly MMN group compared to the daily IFA group (*p*<0.001). There was no change in birth weight or 6-mo-old infant outcome differences between groups when the regression model was adjusted for adherence.

## Discussion

We found that infants born to women receiving twice weekly IFA or MMN during pregnancy did not have different mean birth weights or length-for-age *z*-scores at 6 mo compared to women receiving daily IFA. According to the spread of the 95% CIs, the MD in birth weight and length-for-age *z*-score in infants born to women who received twice weekly compared to daily IFA during pregnancy is smaller than what would be considered clinically important for the infant.

There is a paucity of data on the impact of intermittent antenatal dosing on infant outcomes past the neonatal period. Our study provides important evidence on the impact of prenatal micronutrient supplementation on the first 6 mo of life. We found no significant differences in length-for-age *z*-scores, prevalence of stunting, mean hemoglobin, or prevalence of iron deficiency in infants at 6 mo of age born to women who received intermittent, compared to daily, supplementation during pregnancy. The significance of the difference in head circumference between the twice weekly IFA and the daily IFA group is uncertain and needs to be interpreted with caution, as head circumference at birth was recorded in less than half of the infants.

Infants in the twice weekly IFA group had higher mean cognitive composite scores at 6 mo of age, compared to the daily IFA group (MD 1.91; 95% CI 0.25 to 3.56). This finding needs to be interpreted with caution given the difficulties in assessing cognitive development at this age [36], and long-term monitoring of these infants into childhood is indicated. Currently, there is a paucity of high-quality data available on the effects of iron on cognitive development, and further exploration in this area is urgently required.

Forty percent of women in the daily IFA group were found to have ferritin levels greater than 41 µg/l at 32 wk gestation. Inferior pregnancy outcomes have been associated with higher ferritin levels (>41 µg/l) in the third trimester, and are attributed to increased risk of intrauterine infection, failure of expansion of the maternal plasma volume, and oxidative stress during pregnancy, secondary to a state of temporary iron overload [10]–[13],[20],[37]. Despite the lower ferritin levels in the intermittent supplementation groups, no difference in maternal Hb between groups was observed at 32 wk gestation. Daily IFA supplementation was not associated with hemoconcentration (Hb>130 g/l) in this trial.

WHO recently recommended weekly IFA supplementation for non-anemic pregnant women 18,38. The quality of evidence in the Cochrane review supporting this guideline was low, particularly for birth weight, preterm birth, maternal anemia, and iron deficiency anemia at term, and many of the trials were limited by high risk of selection bias and significant loss to follow-up [19]. In addition, no recommendation was provided for pregnant women in settings where antenatal testing for anemia is not routinely performed. Our large cluster randomised trial provides new evidence to support this guideline, and is of particular significance in Viet Nam, where reports confirm our findings of reduced anemia prevalence in pregnant women to below 20% in many areas, with less than 50% of anemia due to iron deficiency [15]–[17].

Weekly IFA supplementation is also recommended for non-pregnant women of reproductive age in areas with higher rates of anemia, to improve general health as well as improve iron and folate stores pre-pregnancy and in the critical first trimester [33],[39]–[43]. Adoption of an intermittent IFA regimen during pregnancy would allow for integration of long-term IFA supplementation into community-based programs that enable women to take weekly IFA supplements throughout their reproductive years, starting in adolescence and doubling the dose when pregnant [44],[45]. This would potentially improve adherence and availability of supplements, lower costs, and increase coverage of antenatal IFA. In low-income countries where provision of free antenatal IFA supplements is no longer an option, this approach may be particularly effective.

MMN supplementation during pregnancy has potential advantages in Viet Nam, where micronutrient deficiencies (iodine, zinc, and vitamin B12) remain prevalent [15]. In our trial we found a high prevalence of iodine (93%) and vitamin D deficiency (18%) among women in late pregnancy. To date, it is unclear whether the reduced dosage of MMNs delivered via an intermittent regime is too low to correct deficiencies in pregnant women in resource-limited settings. Our trial addresses this important question of the use of intermittent MMN supplementation during pregnancy, and demonstrates no comparative advantage in birth or infant outcomes over daily IFA.

Our findings should be considered within the context of the strengths and limitations of this trial. To our knowledge, ours is the largest randomised controlled trial in a developing setting to investigate the use of intermittent antenatal micronutrient regimes on infant outcomes past the neonatal period, and the first to compare daily IFA with intermittent MMN supplementation during pregnancy. Other strengths were that we worked closely with the local health system and trained commune health workers, resulting in a low refusal rate and low rate of loss to follow-up.

A limitation of our study was that we investigated several outcome measures, and therefore the association that we observed of lower mean cognitive score in infants born to women receiving daily IFA, compared to twice weekly IFA, may be due to type I error. Nevertheless, this finding is of a magnitude of clinical importance, and requires further investigation. Several outcome measures also had a low prevalence, and therefore the trial may have been underpowered to detect differences in these outcomes in the different supplement groups.

Additional limitations included a higher loss to follow-up for the primary outcome of birth weight in the daily IFA arm. Reassuringly, baseline maternal characteristics of mothers of infants who did not have infant birth weight measured did not differ from those of women whose infants did. As blinding was not possible in the daily IFA group, interviews were structured and we attempted to minimise bias by not informing participants and commune health staff about the schedule in other arms of the study. Measurement of head circumference was recorded in only 47% infants. Although commune health workers were trained to record this measurement at every delivery, it is likely that the addition of this non-routine measurement was difficult to implement in commune health stations, where staffing may be limited.

Our chosen setting in a rapidly developing rural area is representative of many parts of Viet Nam, and our findings may also be of relevance to other low- and middle-income countries undergoing economic transition, where improved nutrient intake is reducing the prevalence of iron deficiency anemia. However, it is important to consider that our findings may not extend to the mountainous regions of Viet Nam, or other areas where socioeconomic standards remain low and iron deficiency anemia prevalence is higher [16].

In conclusion, we have shown that twice weekly antenatal IFA or MMN supplementation in an area of Southeast Asia with low anemia prevalence did not produce a clinically important difference in birth weight or infant growth outcomes, compared to daily antenatal IFA. Our finding of a significant improvement in infant cognitive outcome at 6 mo of age following twice weekly antenatal IFA supplementation requires further exploration, and provides additional support for the use of intermittent over daily antenatal IFA regimes in populations with low rates of iron deficiency.

## Supporting Information

Text S1 **Trial protocol.**
(DOC)

Text S2 **CONSORT statement.**
(DOC)

Text S3 **Ethics approval.**
(PDF)

Table S1 **Baseline characteristics of mothers of infants who had birth weight measured compared to those who were lost to follow-up.**
(DOCX)

Table S2 **Maternal and neonatal outcomes with mean difference or odds ratio for comparison of the intervention groups, with and without adjustment for potential confounders.**
(DOCX)

Table S3 **Infant growth and developmental outcomes at 6 months of age, with mean difference or odds ratio, for comparison of the intervention groups.**
(DOCX)

## Abbreviations

BSID III: *Bayley Scales of Infant and Toddler Development, 3rd edition*
CI: confidence interval
GM: geometric mean
Hb: hemoglobin concentration
ICC: intra-cluster correlation coefficient
IFA: iron–folic acid
MD: mean difference
MMN: multiple micronutrient
SD: standard deviation
WHO: World Health Organization
