# Supplementary material for: The Effect of Intermittent Antenatal Iron Supplementation on Maternal and Infant Outcomes in Rural Viet Nam: A Cluster Randomised Trial
Source: PLoS Med. 2013 Jun 18;10(6):e1001470. doi: 10.1371/journal.pmed.1001470 (PMC3708703; doi:10.1371/journal.pmed.1001470)
Supplement: Text S1 — Trial protocol. (DOC) [file pmed.1001470.s001.doc]

| Project Protocol |
| --- |
| Principal Investigator Associate Professor Beverley-Ann Biggs |
| Project name  Does weekly iron supplementation increase iron uptake in pregnant women and improve maternal and infant health? |
| **Lay Abstract** |
|  |

Anaemia and its main cause, iron deficiency, are common in pregnant women in developing countries and adversely affect infant and child health. As well as increasing maternal mortality, maternal iron deficiency anaemia is associated with premature birth, and low birth weight, which in turn is associated with childhood stunting. Children born to iron deficient mothers are more likely to be iron deficient, with increased risks for impaired intellectual development and poorer school outcomes in adolescence. Recently, small studies suggest that iron deficiency in a mother may also increase rates of postpartum depression, which may lead to reduced maternal cognitive functioning and sensitivity and responsiveness to her baby. The infants of depressed mothers in these settings are more likely to be stunted and underweight and to have diarrhoeal diseases and less likely to be completely immunized than infants whose mothers are not depressed. Daily iron, together with folic acid, is recommended by the World Health Organization for pregnant women but often not taken because of unpleasant side-effects, or variable availability. Better strategies are needed to maintain iron stores and decrease anaemia rates during pregnancy to ensure the best health outcomes for mothers and their infants.

Current guidelines in Vietnam recommend iron-folic acid supplementation in pregnancy taken daily. However supply of tablets is variable and compliance is poor and up to 60% of women become anaemic during pregnancy. Weekly iron-folic acid supplementation for pregnant women is a possible alternative to daily supplements and has been shown to be effective in small research studies. Multiple micronutrient supplements (MMN), which contain iron and other micronutrients, are another option for use in pregnancy. A recent study in China showed that MMN given daily in pregnancy resulted in increased infant birth weight, but a slightly higher rate of prematurity when compared to daily iron-folic acid supplementation.

We have shown in a large population-based program that weekly iron-folic acid supplementation reduces anaemia rates in non-pregnant Vietnamese women by 52% over 12 months. A weekly regime of iron-folic acid or MMN supplementation started in adolescence and continued throughout the reproductive years, including in pregnancy, may result in better compliance by women, fewer side-effects and an easier administrative system, leading to increased availability of supplements to pregnant women. However, more information is needed about the use of intermittent supplementation in pregnancy with both iron-folic acid and MMN.

In this study, we will compare the effects of three different methods of iron supplementation in 1224 pregnant Vietnamese women on maternal haemoglobin and iron stores, infant birth weight, and infant anaemia, infant height, weight and developmental screening during the first 12 months of life. Maternal depression will be correlated with infant growth and development, after controlling for maternal iron status. Prior to the study we will consult widely with provincial health officials, community leaders, health workers and community members to ensure high levels of community participation in the project. All pregnant women who are <20 weeks gestation during the recruitment period will be invited to participate and will be assigned to one of three study arms depending on the commune they live in. Women will take either (i) two capsules of iron-folic acid per week (60 mg elemental iron/capsule), or (ii) one capsule of iron-folic acid per day (60mg iron/capsule), or (iii) two capsules of MMN per week (60mg iron/capsule). Women will have their height and weight measured at the third trimester, and at 6 months postpartum, as well as having blood tests to measure haemoglobin, iron stores and other micronutrients. Infant birth details will be recorded, and infant growth and development will be assessed at 6 months after birth. Infants will be checked for anaemia and iron deficiency at 6 months.

Vietnam is currently considering a nationwide weekly iron-folic acid (or MMN) supplementation program for non-pregnant women of reproductive age. Our results will clarify whether this is also the best approach for use when women become pregnant. If so, women could use weekly iron supplementation throughout their reproductive years, including during pregnancy. The practical outcome will be new information about the clinical impact of different iron supplementation approaches on maternal and infant health. More broadly this research has the potential to vastly increase compliance with and improve the distribution of iron supplements in pregnancy, which will lead to improved effectiveness with reduced rates of iron deficiency and anaemia, increased birth weight, and improved infant growth and development and in the long term school performance.

| Principal Investigator Associate Professor Beverley-Ann Biggs |
| --- |
| **Scientific Abstract** |
|  |

Iron deficiency anaemia is widely prevalent in pregnant women despite global and national policies for daily iron supplementation in most countries. This is because of variable availability of supplements at the primary level, and poor compliance due to unpleasant side-effects. Maternal iron deficiency has serious repercussions for maternal and infant health, and is linked with increased risk of maternal death, preterm delivery, intrauterine growth retardation, low birth weight, and impaired infant growth and development. Preliminary evidence suggests that iron deficiency may also be a cause of maternal depression in the perinatal period, which may impact adversely on fetal growth and infant development in the early years. WHO advocate iron and folic acid supplementation taken once a week for non-pregnant women living in areas with high rates of anaemia. We have demonstrated that a population-based weekly iron folic-acid supplementation program for non-pregnant women in North Vietnam reduced anaemia rates by 50% over a 30-month period ^[1, 2]^. Small studies have shown that weekly supplements are also effective in maintaining haemoglobin (Hb) in pregnancy, and emerging evidence suggests that small daily doses of iron during pregnancy, as well as weekly or intermittent dosing starting early in pregnancy, are safer and essentially as efficacious as daily iron in preventing iron deficiency and improving iron stores. Multiple micronutrient supplements (MMN), which contain iron and other micronutrients, are another option for use in pregnancy. A recent study in China showed that MMN given daily in pregnancy resulted in increased infant birth weight, but a slightly higher rate of prematurity when compared to daily iron-folic acid supplementation ^[3]^. A weekly regime of iron-folic acid or MMN supplementation started in adolescence and continued throughout the reproductive years, including in pregnancy, may result in better compliance by women, fewer side-effects and an easier distribution system, leading to increased availability of supplements. However, it remains to be determined if weekly iron-folic acid or MMN supplements will be as effective as the recommended daily iron-folic acid when given in a large randomized controlled trial in a resource-constrained country, and whether there will be measurable benefits in maternal haemoglobin and iron stores, birth outcomes and infant growth and development. The Vietnamese Government acknowledges maternal and infant nutrition, and childhood stunting as urgent priorities.

Research Plan

We will conduct a cluster-randomized controlled trial in Hanam province, where there were 11,551 recorded births in 2008. We will randomize 102 communes to one of the three intervention arms of the study, and all women that are pregnant during the recruitment period and less than 16 weeks gestation will be invited to participate. We will recruit 1224 pregnant women who will take either (i) two capsules of iron-folic acid per week (60 mg elemental iron/capsule), or (ii) one capsule of iron-folic acid per day (60mg iron/capsule), or (iii) two capsules of MMN per week (60mg iron/capsule).

Aims 1. To compare the effect of twice weekly provision of iron-folic acid or MMN supplements, with the recommended daily iron-folic acid supplements during pregnancy on maternal and infant outcomes (including the primary outcome of birth weight; and secondary outcomes of maternal haemoglobin and ferritin at 32 weeks, and infant height for age z scores, haemoglobin, ferritin and cognitive developmental scores at 6 months of age).

2. To compare the effect of twice-weekly provision of iron-folic acid supplements with twice weekly MMN during pregnancy on maternal and infant outcomes, using similar outcomes to Aim 1.

Outcomes and Significance: Vietnam is currently considering a nationwide weekly iron-folic acid or MMN supplementation program for non-pregnant women of reproductive age. Evidence from this study will clarify whether this approach is also suitable for use when women become pregnant. This would increase availability of supplements by allowing easier distribution at village level, and would likely reduce side effects compared to the currently recommended (although poorly administered) daily provision of iron-folic acid supplementation. It will extend previous studies by measuring clinical outcomes of different iron supplementation approaches and the impact on infant outcomes.

| Principal Investigator Associate Professor Beverley-Ann Biggs |
| --- |
|  |
|  |
| Specific Aim(s) of the Project |

**Specific Aims**

1. To compare the effect of twice weekly provision of iron-folic acid or MMN supplements, with the recommended daily provision of iron-folic acid supplements during pregnancy on maternal and infant outcomes during pregnancy and the first 6 months of life (including the primary outcome of birth weight; and secondary outcomes of maternal haemoglobin and ferritin at 32 weeks, and infant height for age z scores, haemoglobin, ferritin and cognitive developmental scores at 6 months of age).

2. To compare the effect of twice weekly provision of iron-folic acid supplements with twice weekly MMN during pregnancy on maternal and infant outcomes during pregnancy and the first six months of life, using similar outcomes to Aim 1.

| Principal Investigator: Associate Professor Beverley-Ann Biggs |
| --- |
| Background and Significance |

Maternal and child health are major health priorities in countries in the Western Pacific region and globally ^[4]^. Under-nutrition is the cause of 3.5 million child deaths/year and it is estimated that at least 200 million children in less developed countries are prevented from attaining their optimal developmental potential due to poverty and associated health, nutrition, and psychosocial factors ^[5]^. Four key risk factors have been identified as main contributors to this situation, stunting, inadequate cognitive stimulation, iodine deficiency, and iron deficiency anaemia, and there is an urgent need to address them. Overall infant physical and mental development is increasingly impaired as these risks accumulate ^[5], [6], [7]^ . Maternal iron deficiency anaemia is an important contributor to these risk factors and will be addressed in our study.

Stunting, the most definitive indicator of chronic, rather than acute, under-nutrition, may affect the development of a child from the early stages of conception through to the third or fourth year of life ^[8], [9]^. It is due to a combination of maternal factors causing inadequate foetal growth before birth; and postnatal factors such as extended periods of inadequate food intake, poor dietary quality, or significant illness ^[5]^. Maternal iron deficiency increases risks of intrauterine growth retardation and low birth weight ^[10]^, which are associated with stunting. Infants who experience iron deficiency during the first 6-12 months of life are likely to experience developmental delays in cognitive, social-affective and motor performance that may persist for years, ^[11], [12], [13], [14]^. Maternal anaemia has been shown to be a predictor of iron deficiency in infants ^[15], [16]^. WHO estimate that 42% of pregnant women and 47% of preschool children globally have anaemia, of which 60% is likely to be due to iron deficiency ^[17]^. As well as leading to a reduction in physical and mental capacity, iron deficiency is linked with increased risk of maternal death, preterm delivery, low birth weight, increased neonatal mortality and impaired infant development ^[18], [19], [16]^. Dietary deficiency of bioavailable iron and increased iron demands during pregnancy, blood loss from menstruation and other causes such as intestinal hookworm infection are major contributors to iron deficiency in women in less developed countries ^[18]^. Many women become pregnant with insufficient iron stores to ensure optimal foetal growth and development and satisfactory pregnancy outcomes ^[20]^.

In Viet Nam a daily iron supplementation program for pregnant women has been in operation since in 1993 ^[28]^. The failure of daily iron programs to significantly reduce maternal anaemia is due to limited or variable availability of supplements and poor compliance because of unpleasant gastrointestinal side-effects ^[28]^. Therefore, better strategies are needed to maintain iron stores and decrease anaemia rates during pregnancy to ensure the best health outcomes for mothers and their infants. Weekly provision of iron supplementation for pregnant women may be an effective strategy for overcoming compliance issues related to daily supplementation. ^[29], [30]^. This approach has the advantage of few side-effects and easy distribution at community level, and emerging evidence suggests that small daily doses of iron in pregnancy, as well as intermittent dosing starting early in pregnancy, are safer and essentially as efficacious as daily iron in preventing iron deficiency and improving iron stores[20]. Involving village health workers in encouraging iron supplementation during pregnancy was shown to be effective in reducing anaemia rates in Thailand ^[31]^, and in Vietnam involvement of village health workers in iron distribution may overcome the difficulties that rural women have in accessing appropriate supplementation during pregnancy. Recent evidence also suggests that MMN supplements may be desirable in pregnancy, leading to increased birth weights compared to iron-folic acid ^[3] , [32]^.

In this context, Australian and Vietnamese researchers have formed a partnership to conduct this study among pregnant women in Hanam province, Viet Nam, to investigate the most effective form of iron supplementation during pregnancy on functional maternal and infant outcomes. The research will extend previous studies by measuring clinical outcomes of different iron supplementation approaches on infant health.

A demonstrated effective strategy for improved uptake of antenatal iron supplementation with significant improvements in infant growth and development would have broad implications for policy development in Vietnam and resource-constrained countries in the region. The research, to be conducted in collaboration with WHO, has the potential to rapidly change policy and practices if intermittent iron-folic acid or MMN are shown to be superior, leading to increased compliance, availability and effectiveness of iron supplementation in pregnancy, reduced rates of iron deficiency and anaemia, and improved birth weight, infant growth and development and in the longer term, school performance.

| Principal Investigator Associate Professor Beverley-Ann Biggs |
| --- |
| Supportive Preliminary Data |

Viet Nam initiated a daily iron supplementation program for pregnant women in 1993 ^[28]^. We have recently shown that weekly iron-folic acid supplementation and deworming is a practical and effective way to reduce anaemia and hookworm prevalence in women of reproductive age (Fig 1) ^[35], [2]^.

Figure 1. Change in mean Hb for non-pregnant anaemic and non-anaemic women sampled at baseline, three and 12 months after commencement of a weekly iron-folate intervention and deworming. The prevalence of anaemia fell from 37.5% [CI 30.9, 44.1]) to 19.5% [CI 15.4, 23.7]), and of hookworm infection from 76.2% (CI 68.2-82.8) to 25.2% (CI 20.6-30.1, P<0.001) over the one year study period, ^[1], [2]^.

In those who became pregnant during this study, increased birth weight was observed in the group with access to the weekly iron-folic acid supplementation program compared to controls.

Table 1. Mean birth weight, % low birth weight in neonates of women who became pregnant in districts where a weekly iron-folic acid and deworming program had been operational, compared to those in control districts.

|  | Mean Birth Weight | Low birth weight |
| --- | --- | --- |
| Intervention districts (N 168) | 3135g (95% CI 3080-3190) | 2.9% |
| Control districts (N 295) | 3011g (CI 2960-3062) | 7.1% |
| Difference | +124g (CI 49-199) | -4.2%  RR 0.42 (CI 0.16-1.09) |
| P Value | 0.001 | 0.045 |

In a recent survey in Yen Bai, many women kept taking weekly iron-folic acid supplements during pregnancy if they could not get access to daily supplements through antenatal clinics or privately.

As a result of these findings, we have worked with the Government of Vietnam and WHO to reach all 250,000 non-pregnant women of reproductive age in Yen Bai with weekly iron-folic acid supplementation ^[35]^. One of the key factors in this program has been implementation at village level with community engagement and health services strengthening through education and training of all village health workers in the province, and improved linkages between commune and village level ^[35]^. We are currently participating in the development of a ‘Plan of Action to Accelerate the Reduction of Child Stunting in Viet Nam’ ^[33]^, which includes weekly iron for non-pregnant women on a national scale. The proposed study will build on this initiative by determining if intermittent iron-folic acid or MMN is a more effective approach for iron supplementation for pregnant women when compared to daily supplementation in reducing anaemia, and improving iron status, birth weight and infant growth and development. If successful, this evidence would support continuation of weekly iron-folic acid supplementation in women throughout their reproductive years, starting in adolescence taking 60mg elemental iron once a week and increasing to 120mg iron a week during each pregnancy.

| Principal Investigator Associate Professor Beverley-Ann Biggs |
| --- |
| **Experimental Design and Methodology** |
|  |

**Setting**

Although considerable progress in health indicators has been made in Vietnam in the last decade, there is still a high prevalence of chronic malnutrition and stunting among the under five population and of low birth weight; relatively high maternal and neonatal mortality, mainly in ethnic minorities and in remote areas, and an increasing prevalence of life-style related diseases (including suicide, and mental health disorders). Maternal and infant mortality rates among ethnic groups are much higher than the national averages, social inequalities have increased and infant mortality in the poorest 20% is increasing ^[40]^ .

We will undertake this project in Hanam Province, Viet Nam, which has a population of approximately 820,100 people, with most still living in rural areas and working in subsistence agriculture. The main town is approximately 60 km from Hanoi. There are six districts and 116 communes. The province is relatively small and has a high population density (845.8km^2^ and 968 people/km^2^) ^[41]^ so travel time between communes is quite short. At least 80% of births take place in commune health centers with most others occurring in district hospitals. We chose Hanam province for this project because of its closer proximity to Hanoi, which will make the project easier to implement. Within Vietnam, the project will be hosted by the Research and Training Centre for Community Development (RTCCD), a Vietnamese NGO, who will oversee project implementation in Hanam province and will liaise and collaborate with national and provincial Vietnamese Government authorities, including the National Institute for Nutrition, the National Institute for Malariology, Parasitology and Entomology, the Provincial Health Department and the Provincial Maternal and Child Health Service. An Australian project officer will be located in Vietnam to oversee the project.

**Approach and Methodology**

Design: We propose to conduct a cluster-randomised controlled trial of iron supplementation in pregnant women commenced before 16 weeks gestation. A placebo-controlled trial was not contemplated as we considered it unethical to withhold iron supplementation during pregnancy. The trial will have three intervention arms as follows: (1) one capsule of iron-folic acid (IFA) to be taken twice a week (60mg elemental iron /capsule, 2 capsules per week), or (2) one IFA tablet to be taken daily (60mg elemental iron /tablet; 7 tablets per week), or (3) one capsule of MMN to be taken twice a week (60 mg elemental iron/capsule, 2 capsules per week).

Supplements: We have chosen a local pharmaceutical manufacturer, Nam Ha Pharmaceutical Company, to produce the IFA and MMN supplements for the clinical trial. This company has produced IFA supplements for our Yen Bai WIFS program for the last 4 years and has WHO GMP certification. The supplements will require limited registration by the Ministry of Health for use for research purposes.

The formulation of the three types of supplements is shown in the table below.

**Formulation of Supplements -Amount in each capsule**

| Content | Weekly Iron-folic acid^1^ | Weekly multi-micronutrients ^2^ | Daily iron-folic acid^3^ |
| --- | --- | --- | --- |
| Elemental Iron | 60mg | 60mg | 60mg |
| Zinc |  | 20mg |  |
| Iodine |  | 300µg |  |
| Copper |  | 4mg |  |
| Selenium |  | 130µg |  |
| Vitamin A |  | 1.6mg |  |
| Vitamin B |  |  |  |
| - B1 (thiamine) |  | 2.8mg |  |
| - B2 (riboflavin) |  | 2.8mg |  |
| - niacin |  | 36mg |  |
| - B6 |  | 3.8mg |  |
| - B12 |  | 5.2µg |  |
| - Folic acid | 1.5mg | 1.5mg | 0.4mg |
| Vitamin C |  | 140mg |  |
| Vitamin D |  | 400IU |  |
| Vitamin E |  | 20mg |  |

1. Two capsules to be given each week = 120mg iron, 3mg folic acid/week. These will be identical to the MMN tablets.

2. Two capsules to be given each week = 120mg iron, 3mg folic acid, 40mg zinc + approx 4xRDI of other micronutrients.

3. Seven tablets to be given each week = 420mg iron, 2.8mg folic acid

The MMN will contain similar micronutrients to the UNICEF-recommended UNIMMAP daily supplement ^[43]^, except that we will include 2x the amount of micronutrient in each capsule (as women will only take two capsules a week). This means that women will receive 4/7 of the amount of micronutrients per week that they would have received from a daily UNIMMAPP supplement. The exception to this is folic acid. Each of the supplements will contain 1.5mg of folic acid.

Treatment allocation and recruitment: The study will be undertaken in 5 districts of Hanam Province (and will exclude the main provincial town). The commune was chosen as the cluster unit of randomization to reduce the likelihood of interactions between the intervention groups. Communes agreeing to participate in the study will be randomly assigned to one of the three treatment arms: twice weekly provision of IFA, daily provision of IFA, or twice weekly provision of MMN. Randomization will be performed by an independent statistician using ‘ralloc’ in the statistical program Stata (StataCorp, College Station, TX, USA).

Lists kept at commune level will be used to identify all pregnant women in selected communes and all eligible women will be invited to participate by the research team, in conjunction with local village and commune health workers. In Hanam province we expect approximately 1990 live births per district/year (personal communication PI 4). All women in the trial communes that are <16 weeks gestation during the recruitment period will be considered for inclusion in the study. Women with complicated pregnancies (e.g. twins, diabetes, other medical conditions), or Hb<80 g/L will be excluded from the study and referred for medical treatment.

We expect recruitment to take approximately 8 weeks. However, a flexible approach will be taken and the research team will work closely with provincial counterparts to determine the best strategies for recruitment, which may involve recruiting over a longer time period. We plan to increase the recruitment of women in the first trimester of pregnancy by using village and commune health worker staff to inform women who are recently married or trying to become pregnant about the study, and to encourage them to come for early pregnancy testing. In this way we hope to recruit women from six weeks gestation. Written informed consent will be obtained at enrolment.

Reducing bias by blinding the researchers and participants to the intervention, and by training: The two arms of the trial with intermittent supplementation will be blinded. The supplements will be produced by Nam Ha Pharmaceutical Company, Vietnam, who will undertake the blinding process. The IFA and MMN capsules that will be taken twice a week will be identical and a code will be embossed on each blister pack, with the code known only to the manufacturer and the chairperson of the Data Monitoring and Safety Committee.

The daily intervention cannot be blinded to the participants or the field team. To try to overcome biases we plan to promote the project to participants and commune staff as a study of different supplements for use in pregnancy without explicitly informing participants about the schedule in other arms of the study, in this way minimizing the chance of women believing they are not getting ‘enough’, or getting ‘too much’, iron. We will also make it clear that the optimal amount of iron for use in pregnancy is still in doubt. Training sessions for researchers and health workers will emphasize the importance of being objective in all aspects of the study. We do not expect bias in any of the laboratory measures because the laboratory staff will be unaware of the intervention groups.

We will train the research team and selected commune and hospital staff to make triplicate measurements (e.g. birth weight, maternal and infant anthropometry) and select the middle measurement. A second observer will check the measurements. RTCCD staff will administer the depression scales and will not be blinded to the daily intervention. However these are experienced research staff using standard structured tools and with appropriate training we hope to limit bias in the interviews.

Distribution of the supplements and monitoring: At enrolment, women will receive blister packs containing two month’s supply of intervention. A senior RTCCD project officer and a local provincial project officer will be assigned to each arm of the study for monitoring visits and the senior person will provide ongoing training and supervision to the local staff member through out the project. Each woman will be visited approximately every six weeks to distribute the intervention for the next period. At this visit, the researcher will collect old blister packs and record the number of tablets consumed, as well as seeking information about compliance, side effects and pregnancy complications. Monitors will also encourage women to attend the commune health station for delivery. We expect tracking for follow up to be straightforward because there are relatively low rates of whole household relocation in Vietnam. Retention rates using these strategies have previously been high ^[42]^.

Exclusions and loss to follow-up: A recent observational study in Ha Nam recruited 500 pregnant women of whom none were severely anaemic (Hb <70g/L) and we anticipate this will be similar in this trial. While we have no quantitative data from Hanam on complicated pregnancies, anecdotal reports from the field suggest these are less than 5% of all births. One likely problem will be drop-outs by women who are reluctant to provide a blood sample at 32 weeks. Data from the observational study suggested that this could be as high as 15%.

Ethics: To protect privacy, code numbers and not names will be used. Other ethical considerations are outlined in the Australian National Ethics Application Form which was recently submitted and approved by the Melbourne Health HREC. Amendments will be sought from the HREC for any significant changes to the protocol that need to be made in the planning phase.

Assessment of women will occur at study enrolment, 32 weeks gestation and 6 months postpartum and of infants at birth and 6 months of age as follows:

**Figure 2. Study design**

Communes randomly assigned

Twice weekly multiple micronutrients

Daily iron intervention

Twice weekly iron intervention

Eligible pregnant women <16 weeks, consent, interview, ht, wt, arm circ, Hb, micronutrient tests*. Twice weekly iron commenced.

Six weekly monitoring

Eligible pregnant women <16 weeks, consent, interview, ht, wt, arm circ, Hb, micronutrient tests*. Twice weekly MMN commenced.

Six weekly monitoring

Eligible pregnant women <16 weeks, consent, interview, ht, wt, arm circ, Hb, micronutrient tests* Daily iron commenced.

Six weekly monitoring

32 weeks gestation: interview, anthropometry, Hb, micronutrient tests.

32 weeks: interview, anthropometry, Hb, micronutrient tests.

32 weeks: interview, anthropometry, Hb, micronutrient tests.

Birth weight, length, HC and outcomes

Birth weight, length, HC and outcomes

Birth weight, length, HC and outcomes

6 months Postpartum-interview, maternal anthropometry, Hb, ferritin; infant ht, wt, HC, Hb, ferritin, developmental screening

6 months Postpartum-interview, maternal anthropometry, Hb, ferritin; infant ht, wt, HC, Hb, ferritin, developmental screening

6 months Postpartum-interview, maternal anthropometry, Hb, ferritin; infant ht, wt, HC, Hb, ferritin, developmental screening

* Micronutrients to be tested include ferritin, TfR, folate, vitamin B12.
Hb = haemoglobin, Ht = height, Wt = weight, TfR = transferrin receptor, MMN = multiple micronutrients, HC = head circumference.

Measures: Data will be collected via blood tests and a range of measures including structured individual interviews using psychometric instruments, maternal and infant anthropometry, compliance and side-effects, birth records and assessment of infant growth and developmental screening as described below.

Aim 1. To compare the effect of twice weekly provision of IFA or MMN supplements, with the recommended daily provision of IFA supplements during pregnancy on maternal and infant outcomes during pregnancy and the first 6 months of life (including the primary outcome of birth weight; and secondary outcomes of maternal haemoglobin and ferritin at 32 weeks, and infant height for age z scores, haemoglobin, ferritin and cognitive developmental scores at 6 months of age).

*Rationale: Iron deficiency in pregnancy is associated with increased maternal mortality, preterm delivery, low birth weight* ^[4]^ *and low iron stores in infancy* ^[16]^*. Low birth weight infants have higher mortality rates, and are more likely to be stunted. Stunting is a good indicator of later poor performance at school* ^[44]^*. Iron deficiency in infancy may also contribute to poor cognitive development and later school performance* ^[4]^, ^[45]^*.* *Iron deficiency anaemia is widely prevalent in pregnant women despite global and national policies for daily provision of iron supplementation in most countries. This is thought to be because of poor availability of supplements at the primary level, and poor compliance because of unpleasant side-effects. On the other hand, weekly iron supplementation has been shown to be effective in improving iron status in non-pregnant women in Vietnam and elsewhere, and in small studies in pregnant women. This approach has the advantage of few side-effects and easier distribution. Furthermore, emerging evidence suggests that small daily doses or iron during pregnancy, as well as weekly dosing starting early in pregnancy, are safer and essentially as efficacious as daily iron in preventing iron deficiency and improving iron stores* ^[20]^*. Daily provision of MMN supplementation may also have advantages over daily IFA- as Zheng et al recently showed increased birth weight with their use in pregnant women in China* ^[3]^*. It remains to be determined if weekly provision of IFA or MMN will be superior to daily provision of IFA, when given in a large cluster-randomized controlled trial in a developing setting, using infant birthweight as the primary outcome.*

Maternal socio-demographic factors – will be measured in one-to one interviews using previously developed and pilot-tested questions. These interviews will occur after obtaining signed informed consent, and will be conducted by experienced RTCCD staff that have had refresher training as mentioned above. For women receiving a twice weekly supplement the researchers will be blinded to the intervention.

Maternal anaemia and micronutrient deficiencies: In women who agree, venipuncture blood samples will be collected at enrolment, 32 weeks gestation and 6 months postpartum by staff trained in the procedure. Hb will be measured in the field using HemoCue, and serum samples frozen and transported to Hanoi for testing at the National Institute of Nutrition (or accredited laboratory in Australia or US – depending on cost) for iron indices and other micronutrient indicators (serum ferritin^61^, transferrin receptor, folate, vitamin B12) using standard laboratory methodology. Laboratory staff will be not be aware of the intervention being taken by a participant or any other study data.

Maternal and Infant anthropometry Maternal anthropometry will be assessed by mid-upper arm circumference, body weight and height in all participants at enrolment, 32 weeks gestation, and, six months postpartum This provides a portable, universally applicable, inexpensive and non-invasive technique for assessing body composition and reflects both health and nutritional status ^[46]^. Mid-upper arm circumference will be measured at the midpoint between the acromion and the olecranon process. The arm circumference will be recorded to the nearest 0.1cm. Mother's height will be measured with a portable stadiometer (Seca 214, Hamburg, Germany) and weight with a mother-infant scale (Seca 872, Hamburg, Germany).

All commune health centers involved in the study will be provided with new tabletop digital infant weighing scales that measure to 10 grams (supplied by BF20510, LAICA, Italy) as existing scales do not have the required sensitivity. In the event of a home birth, commune health workers will be advised to measure and weigh the baby as soon as possible after birth (at least within 3 days), either at the commune health station or in the home.

Measurements of child length (crown-heel) will be made using a portable infantometer (Seca 210, Hamburg, Germany), mother's height with a portable stadiometer (Seca 214, Hamburg, Germany), child's weight with a mother-infant scale (Seca 872, Hamburg, Germany). Growth (height-for-age, weight-for-age, and weight-for-length) will be evaluated using the World Health Organization Child Growth Standards 2006^[47]^. Head circumference will be measured with a non-stretchable tape, using standard procedures.

Standardization of anthropometric measurements: Trained members of the research team will make the maternal and infant anthropometric measurements. A nominated commune health worker and birth attendant will measure birthweights in each commune in the study. These staff will be trained using WHO and CDC resources^[48-50]^. Nominated labor ward staff in the five district hospitals will be included in these training sessions. Each measurement will be made in triplicate by two observers and the middle value used. In addition the monitors and field supervisors will provide ongoing support for commune staff and ensure that scales and measurements are standardized across all sites.

Compliance and side-effects – each participant will be visited by a member of the research team every six weeks, who will collect old blister packs and record the number of supplements that have been removed from the pack. They will ask about compliance, side-effects and any other health issues, as well as providing new supplements.

Infant anaemia and iron deficiency

If mothers agree, venipuncture blood samples will be collected from infants at 6 months of age and Hb and iron stores (ferritin) will be measured as above.

Infant development screening Advice from researchers with experience in this area (Lourdes Schnaas – Mexico) and a review of the literature suggests that a comprehensive assessment using the Bayley Scales of Infant Development III (BSID) is the best approach in this situation ^[51]^. This will provide an infant development score that can be compared between the study groups, and the assessment will take 30-45 minutes. The BSID has language, cognitive, social-emotional, motor and adaptive behavior (caregiver report) subscales that can be scored separately, so that domain-specific assessments can be made, providing comprehensive developmental screening. We plan to recruit local psychologists and paediatricians with experience in developmental screening and provide them with training in the use of the BSID using either local or international experts. The instrument will be pilot tested in Hanam province and adapted to the local cultural context. The infant assessment team will be additional to the core research team and will not have access to other maternal or infant data, including iron status or maternal depression scores, thereby limiting bias.

Aim 2. To compare the effect of twice-weekly provision of IFA supplements with twice-weekly MMN during pregnancy on maternal and infant outcomes during pregnancy and the first six months of life, using similar outcomes to Aim 1.

*Rationale:* *Daily MMN in pregnancy have been shown in some studies to have advantages over daily IFA, especially in terms of birth weight* ^[3,32]^*. Similarly, twice-weekly MMN may be slightly more efficacious than twice-weekly IFA as micronutrients such as vitamin C may increase iron absorption, and folic acid, vitamin B12 and vitamin A may improve Hb levels. However, the presence of zinc in the MMN supplements may lead to a reduction in iron absorption* ^[52]^. *It remains to be determined* if there are any significant differences in clinical outcomes between the twice weekly provision of IFA and MMN.

We will compare outcomes in the twice weekly IFA and MMN arms of the trial. However, we expect any differences in outcomes to be small given that both formulations contain 60mg elemental iron. We will use similar analysis techniques for Aim 1. Estimates (95% confidence intervals) of the difference in mean haemoglobin levels at 32 weeks, ferritin levels at 32 weeks, birthweight and height for age (at 6 months of age) between the two weekly trial arms will be derived using linear mixed-effects modeling incorporating a random effect for commune to allow for clustering. For the outcomes haemoglobin and ferritin, the statistical analysis will adjust for the baseline measurement.

**Table 1: Schedule of administration of maternal and infant assessments**

**1. Mothers**

| **Measure** | **Enrolment <16 wks gestation** | **32 weeks gestation** | **6 months postpartum** |
| --- | --- | --- | --- |
| HemaCue test for Hb | √ | √ | √ |
| Venipuncture blood sample | √ | √ | √ |
| Maternal anthropometry (ht, wt, Mid upper arm circumference) | √ | √ | √ |
| Study-specific interview | √ | √ | √ |

**2. Infants**

| **Measure** | **Birth** | **6 months of age** |
| --- | --- | --- |
| Birth assessment | √ |  |
| Infant anthropometry (wt, length, HC) | √ | √ |
| Developmental Screening using Bayley Scales |  | √ |
| HemaCue test for Hb |  | √ |
| Venipuncture blood sample |  | √ |

Sample size calculations

Hb data from pregnant women in Hanam province on which to base our sample size estimates and data regarding the number of pregnant women available for recruitment has recently been collected in an observational study. There were on average 10 eligible pregnant women of 12 to 20 weeks gestation per commune (mean 10, range 2-20). In the proposed study all eligible pregnant women in communes allocated to each arm of this study will be invited to receive the intervention.

This cluster randomized trial will recruit 34 communes / 408 pregnant women per treatment arm (i.e. a total of 1224 women from 102 communes) where the average commune size is 12 women per commune and it is expected that 5% of women will be excluded due to pregnancy complications and 15% will be lost to follow-up.

With 95% confidence the above sample size has at least 80% statistical power to detect clinically important differences of 6 g/L for haemoglobin (power = 98%, SD=12g/L, ICC=0.12)[[1]](#footnote-2), 100g for birthweight (power = 81%, SD= 389g, ICC=0.03)[[2]](#footnote-3), and 0.3 for the height for age Z-score (power = 93%, SD=0.9, ICC=0.05)[[3]](#footnote-4). The above sample size calculations were performed using ‘sampsi’ and ‘sampclus’ in the statistical program Stata (StataCorp, College Station, TX, USA).

Our primary hypotheses (and sample size estimates) are based on continuous measurements to ensure that all information is included. The optimal Hb in late pregnancy is not clear as there is evidence that moderately low and higher haematocrits may impair birth outcomes. We are also interested in the absolute mean change of ferritin at 32 weeks, as this will reflect the effectiveness of the intervention in maintaining womens’ iron stores into the third trimester of pregnancy. This approach may reduce our power to asses change in percentage of women with anaemia or iron deficiency.

Data Management

Data will be recorded on paper forms and entered into a customized database by a trained data entry officer in the provincial office. It will be checked and cleaned by an epidemiologist in the Hanoi RTCCD office. The database will be developed in conjunction with Associate Professor Michael Dibley (University of Sydney) and scientists at the National Institute for Nutrition who were involved in the Zeng et al. (2008) study in China. Stata (vs10) will be used for data analysis.

Statistical Analysis

Dr Julie Simpson (Centre for Molecular, Environmental and Genetic Analytic Epidemiology, Melbourne School of Population Health, University of Melbourne) will oversee statistical analysis of trial data ^[58]^. Estimates (95% confidence intervals) of the difference in mean haemoglobin levels at 32 weeks, ferritin levels at 32 weeks, birthweight and height for age (at 6 months of age) between the daily and weekly trial arms will be derived using linear mixed-effects modeling incorporating a random effect for commune to allow for clustering. For the outcomes haemoglobin and ferritin, the statistical analysis will adjust for the baseline measurement. Since the distribution of ferritin levels are typically skewed, these levels will be log_e_ transformed before analysis and the comparison of levels between trial arms presented as the ratio of the geometric means.

All statistical analyses will be performed using the statistical program Stata, Version 10 (StataCorp, College Station, TX, USA).

**Table 2: Project timeline**

| **Task** | **Year 1** | | | | **Year 2** | | | | **Year 3** | | | |
| --- | --- | --- | --- | --- | --- | --- | --- | --- | --- | --- | --- | --- |
| PI 1, 2 & 4 to set up study in VN |  |  |  |  |  |  |  |  |  |  |  |  |
| Planning, development of data forms, database, project documentation |  |  |  |  |  |  |  |  |  |  |  |  |
| Appoint new staff |  |  |  |  |  |  |  |  |  |  |  |  |
| Ethics approvals |  |  |  |  |  |  |  |  |  |  |  |  |
| Interviewer training |  |  |  |  |  |  |  |  |  |  |  |  |
| Community mobilization and health worker training |  |  |  |  |  |  |  |  |  |  |  |  |
| Recruit and assess pregnant women |  |  |  |  |  |  |  |  |  |  |  |  |
| PI 1&2 visit VN to review progress |  |  |  |  |  |  |  |  |  |  |  |  |
| Late pregnancy assessment |  |  |  |  |  |  |  |  |  |  |  |  |
| Data Monitoring and Safety Committee review meeting |  |  |  |  |  |  |  |  |  |  |  |  |
| Assess mother/infant (6 weeks) |  |  |  |  |  |  |  |  |  |  |  |  |
| Assess mother/infant (6 months) |  |  |  |  |  |  |  |  |  |  |  |  |
| PI 3 to visit VN to review progress |  |  |  |  |  |  |  |  |  |  |  |  |
| Data cleaning and analysis |  |  |  |  |  |  |  |  |  |  |  |  |
| PI 4, -data analysis in Melbourne |  |  |  |  |  |  |  |  |  |  |  |  |
| Write report/recommendations English &VN |  |  |  |  |  |  |  |  |  |  |  |  |
| Distribute report at final meeting |  |  |  |  |  |  |  |  |  |  |  |  |
| Prepare journal articles |  |  |  |  |  |  |  |  |  |  |  |  |

**Outcomes and Significance:**

This project will have direct benefits for the women and children of Hanam province as it will strengthen the health system by working with community leaders and village health workers to inform women about the importance of iron through the life cycle and prevention of iron deficiency, as well as training village and commune health workers about anaemia, iron deficiency, iron requirements in pregnancy, standardized cord clamping and exclusive breast-feeding. We will also work with provincial and district leaders to ensure that the benefits of iron supplementation programs are well understood and encourage planning for future sustainable initiatives to provide IFA or MMN supplementation to women of reproductive age. Our choice of a local pharmaceutical manufacturer, Nam Ha Pharmaceutical Company, to produce the IFA and MMN supplements for the clinical trial will facilitate the availability of IFA and MMN supplements as, if the study is successful, it will be straightforward for the manufacturer to fully register the supplements for sale in Vietnam, thereby increasing their availability and affordability through both the public and private sectors.

Nationally, Vietnam is currently considering a weekly IFA supplementation program for non-pregnant women of reproductive age in provinces with moderate and high rates of anaemia. Weekly MMN is also being considered as an alternative for this program even though it would be a substantially higher cost. Evidence from this study will clarify whether this approach is also suitable for use when women become pregnant. If so, women would be able to commence weekly IFA or MMN supplementation in adolescence, and continue taking the supplement through the reproductive years, simply doubling the dose for the duration of each pregnancy. This would have the practical benefits of being more economical, and allowing a distribution system to be established at the primary level through existing community organizations. This approach is supported by our previous work where we found that distribution of blister packs of supplements through village health workers on a monthly basis resulted in compliance of >80% in non-pregnant women over 30 months (BB, unpublished data).

The results from the proposed study will extend previous studies by measuring clinical outcomes in mothers as well as in their infants. The finding that intermittent provision of IFA or MMN supplements is the best approach to achieving optimal iron stores during pregnancy and improved infant outcomes would have the potential to vastly increase compliance, effectiveness and availability of iron supplementation in pregnant women in Vietnam and other resource-constrained countries. Whether women access iron supplementation before pregnancy through a free government-sponsored program delivered by village health workers, or through local cost recovery mechanisms ^[28]^, they could continue to access the same supplements from the same source (but double dose) once they become pregnant. This would positively affect availability and compliance, leading to reduced rates of iron deficiency and anaemia, and improvements in birth weight, infant growth and development and, in the longer term, child health.

| Principal Investigator Associate Professor Beverley-Ann Biggs |
| --- |
| **Literature References** |

| Principal Investigator Associate Professor Beverley-Ann Biggs |
| --- |
| **Collaborative Arrangements** |

This project is a collaboration between the Department of Medicine and Key Centre for Women’s Health at the University of Melbourne(UOM), the Murdoch Childrens Research Institute, Melbourne, Australia and the Research and Training Centre for Community Development, Hanoi Vietnam (RTCCD). As supervising agency UOM will enter a research collaboration agreement with RTCCD to implement the project, in conjunction with a UOM project manager who will be based in Hanam Province where the project is to be conducted. The collaboration agreement will comply with the terms and conditions imposed by the University of Melbourne for the conduct of the research and will specify the timeline, budget and payment process for project implementation.

**Programmatic arrangements**

General oversight will be provided by the Principal Investigator working with the co-principal investigators. However, RTCCD will be responsible for arrangements within Vietnam for project implementation including contracting local institutes - the National Institute for Malariology, Parasitology and Entomology and the National Institute for Nutrition for serum collection and sample analysis respectively (unless otherwise agreed). RTCCD will undertake enrollment of participants, conduct qualitative surveys and testing, preliminary data analysis and reporting. The UOM project manager will work closely with RTCCD in Hanam province in the development of a detailed project implementation document, and will provide support and oversight during field activities. He will also ensure that ethical standards are understood and integrated into planning, and that data collection and management is rigorous. He will also liaise widely with stakeholders, including UNICEF, WHO, NGOs, the Ministry of Health, to promote the project and disseminate results. Programmatic arrangements are further described in the Experimental Design and Methodology section

**Administrative arrangements**

Administrative and management services will be shared between Melbourne and Vietnam. A project office will be established in Phu Ly, Hanam province from which field research activities will be managed by the RTCCD team leader under direction by from national team based at the project office and at RTCCD offices in Hanoi and supported by an experienced expatriate Project Manager and researcher. The dedicated project team will work with counterparts in national and provincial health authorities to ensure smooth implementation.

1. Based on data from observational study in Hanam province of pregnant women from 12 to 22 weeks gestation (Tran, unpublished). [↑](#footnote-ref-2)
2. Based on data collected in Yen Bai province, Vietnam (Passerini, unpublished) and Intraclass correlation (ICC) based on Zeng L, Cheng Y, Dang S et al. BMJ 2008;337:a2001. [↑](#footnote-ref-3)
3. Standard deviation based on Baqui AH, Zaman K, Persson LA et al. J Nutr, 133:4150-4157,2003. [↑](#footnote-ref-4)
