# Supplementary material for: The Effect of Intermittent Antenatal Iron Supplementation on Maternal and Infant Outcomes in Rural Viet Nam: A Cluster Randomised Trial
Source: PLoS Med. 2013 Jun 18;10(6):e1001470. doi: 10.1371/journal.pmed.1001470 (PMC3708703; doi:10.1371/journal.pmed.1001470)
Supplement: Table S1 — Baseline characteristics of mothers of infants who had birth weight measured compared to those who were lost to follow-up. (DOCX) [file pmed.1001470.s004.docx]

Table S1. Baseline Characteristics of mothers of infants who had birthweight measured, compared to those who were lost to follow up.

|  |  |  |
| --- | --- | --- |
| **Characteristic** | **Mothers of infants who had birthweight measured ^1^** | **Mothers of infants who were lost to follow up^1^** |
|  |  |  |
| Maternal age (years) | 26.8 [5.0] | 28.6 [5.8] |
| Mean age [SD] |  |  |
| <20 | 50/1258 (4.0) | 5/90 (5.6) |
| 20-24 | 400 /1258(31.8) | 17/90 (18.9) |
| 25-29 | 497 /1258 (39.5) | 32/90 (35.6) |
| 30-34 | 206 /1258 (16.4) | 21/90 (23.3) |
| ≥35 | 105/1258 (8.4) | 15/90 (16.7) |
| Maternal education |  |  |
| Primary school (up to Year 5) | 198/1258 (15.7) | 21/90 (23.3) |
| Secondary school (Years 6 – 9) | 628/1258 (49.9) | 40/90 (44.4) |
| High school (Years 10 – 12) | 307/1258 (24.4) | 19/90 (21.1) |
| Post-secondary qualification | 125/1258 (9.9) | 10/90 (11.1) |
| Employment |  |  |
| Farmer | 513/1258 (40.8) | 41/90 (45.6) |
| Factory worker | 182/1258 (14.5) | 8/90 (8.9) |
| Trader | 237/1258 (18.8) | 19/90 (21.1) |
| Government official | 140/1258 (11.1) | 15/90 (16.7) |
| Clerk | 46/1258 (3.7) | 1 (1.1) |
| Not in income-generating work | 122/1258 (9.7) | 5/90 (5.6) |
| Other | 18/1258 (1.4) | 1/90 (1.1) |
| Gravidity |  |  |
| 0 | 386/1258 (30.7) | 21/90 (23.3) |
| 1 | 450/1258 (35.8) | 27/90 (30) |
| 2-3 | 363/1258 (28.9) | 35/90 (38.9) |
| 4-5 | 54/1258 (4.3) | 6/90 (6.7) |
| ≥6 | 5/1258 (0.4) | 1/90 (1.1) |
| Previous stillbirth or early neonatal death | 57/1258 (6.5) | 6/90 (8.7) |
| Mean maternal weight (kg) [SD] | 46.9 [5.4] | 46.9 [5.7] |
| Mean maternal height (cm) [SD]^2^ | 153.6 [4.8] | 153.4 [4.6] |
| Mean mid upper arm circumference (cm)[SD] | 23.9 [2.95] | 24.1 [2.3] |
| Mean body mass index (kg/m^2^) [SD]^2^ | 19.9 [1.99] | 19.9 [2.18] |

^1^ Values are mean [SD], or number (%)

^2^ Data on height missing for 1 woman
