# Supplementary material for: The Effect of Intermittent Antenatal Iron Supplementation on Maternal and Infant Outcomes in Rural Viet Nam: A Cluster Randomised Trial
Source: PLoS Med. 2013 Jun 18;10(6):e1001470. doi: 10.1371/journal.pmed.1001470 (PMC3708703; doi:10.1371/journal.pmed.1001470)
Supplement: Table S2 — Maternal and neonatal outcomes with mean difference or odds ratio for comparison of the intervention groups, with and without adjustment for potential confounders. (DOCX) [file pmed.1001470.s005.docx]

Table S2. Maternal and neonatal outcomes with mean difference or odds ratio for comparison of the intervention groups, with and without adjustment for potential confounders.

| **Maternal and neonatal outcomes** | **Mean difference/Odds Ratio/Geometric Mean Ratio (95% CI)^1^** | **P value** | **Mean difference/Odds Ratio/Geometric Mean Ratio (95% CI)^2^** | **P value** |
| --- | --- | --- | --- | --- |
| **Birthweight (grams)** |  |  |  |  |
| Daily IFA | Reference |  | Reference |  |
| Twice weekly IFA | 28.0 (-22.1 to 78.1) | 0.27 | 32.5 (-16.3 to 81.3) | 0.19 |
| Twice weekly MMN | -36.8 (-81.9 to 8.2) | 0.11 | -19.7 (-69.7 to 30.3) | 0.44 |
| **Hemoglobin(g/L) at 32 weeks^3^** |  |  |  |  |
| Daily IFA | Reference |  | Reference |  |
| Twice weekly IFA | 0.02 (-2.06 to 2.10) | 0.99 | 0.03 (-2.04 to 2.09) | 0.98 |
| Twice weekly MMN | -1.02 (-3.55 to 1.52) | 0.43 | -1.07 (-2.04 to 2.10) | 0.40 |
| **Ferritin (µg/L) at 32 weeks^3^** |  |  |  |  |
| Daily IFA | Reference |  | Reference |  |
| Twice weekly IFA | 0.73 (0.67 to 0.80) | <0.001 | 0.72 (0.67 to 0.79) | <0.001 |
| Twice weekly MMN | 0.62 (0.57 to 0.68) | <0.001 | 0.61 (0.56 to 0.67) | <0.001 |
| **Infant length for age z scores** |  |  |  |  |
| Daily IFA | Reference |  | Reference |  |
| Twice weekly IFA | -0.13 (-0.28 to 0.02) | 0.09 | -0.14 (-0.29 to 0.01) | 0.07 |
| Twice weekly MMN | -0.04 (-0.20 to 0.12) | 0.62 | -0.05 (-0.20 to 0.10) | 0.54 |
| **Infant hemoglobin (g/L)** |  |  |  |  |
| Daily IFA | Reference |  | Reference |  |
| Twice weekly IFA | -0.55 (-2.44 to 1.33) | 0.56 | -0.58 (-2.52 to 1.35) | 0.55 |
| Twice weekly MMN | 0.75 (-1.26 to 2.76) | 0.46 | 0.74 (-1.31 to 2.78) | 0.48 |
| **Infant cognitive development** |  |  |  |  |
| Daily IFA | Reference |  | Reference |  |
| Twice weekly IFA | 1.91(0.25 to 3.56) | 0.02 | 1.83 (0.18 to 3.50) | 0.03 |
| Twice weekly MMN | 0.78 (-0.76 to 2.33) | 0.32 | 0.76 (-0.77 to 2.29) | 0.33 |

^1^ Model adjusted for cluster randomisation

^2^Model has been adjusted for maternal age, parity, and cluster randomisation.

In addition to the these variables, birthweight has also been adjusted for infant gender and gestational age.

^3^ Baseline values included in model
