# Supplementary material for: The Effect of Intermittent Antenatal Iron Supplementation on Maternal and Infant Outcomes in Rural Viet Nam: A Cluster Randomised Trial
Source: PLoS Med. 2013 Jun 18;10(6):e1001470. doi: 10.1371/journal.pmed.1001470 (PMC3708703; doi:10.1371/journal.pmed.1001470)
Supplement: Table S3 — Infant growth and developmental outcomes at 6 months of age, with mean difference or odds ratio, for comparison of the intervention groups. (DOCX) [file pmed.1001470.s006.docx]

Table S3. Infant growth and developmental outcomes at 6 months of age, with mean difference or odds ratio, for comparison of the intervention groups.

| **Infant growth outcomes at 6 months of age** | **Mean [SD] or Number (%)** | **Mean difference or Odds Ratio**  **(95% CI)^1^** | **P value** | **ICC** |
| --- | --- | --- | --- | --- |
| **Length** |  |  |  | 0.006 |
| Daily IFA | 66.1 [2.3] | Reference |  |  |
| Twice weekly IFA | 65.8 [2.1] | -0.31 (-0.68 to 0.07) | 0.11 |  |
| Twice weekly MMN | 66.0 [2.4] | -0.11 (-0.48 to 0.26) | 0.55 |  |
| **Length for age z scores** |  |  |  |  |
| Daily IFA | 348/351 (99.1) | Reference |  | 0.005 |
| Twice weekly IFA | 361/363 (99.4) | -0.13 (-0.28 to 0.02) | 0.10 |  |
| Twice weekly MMN | 329/335 (98.2) | -0.04 (-0.19 to 0.12) | 0.62 |  |
| **Stunting** |  |  |  | 0.02 |
| Daily IFA | 23/348 (6.6) | Reference |  |  |
| Twice weekly IFA | 22/361 (6.1) | 0.92 (0.47 to 1.80) | 0.80 |  |
| Twice weekly MMN | 23/329 (7.0) | 1.06 (0.55 to 2.05) | 0.86 |  |
| **Infant developmental outcomes at 6 months of age** |  |  |  |  |
| **Language** |  |  |  |  |
| Daily IFA | 94.0 [8.04] | Reference |  | 0.01 |
| Twice weekly IFA | 94.8 [8.2] | 0.80 (-0.52 to 2.12) | 0.23 |  |
| Twice weekly MMN | 94.8 [7.7] | 0.80(-0.55 to 2.16) | 0.24 |  |
| **Motor** |  |  |  |  |
| Daily IFA | 112.3 [18.2] | Reference |  | 0.0006 |
| Twice weekly IFA | 113.4 [19.0] | 1.17 (-1.41 to 3.76) | 0.37 |  |
| Twice weekly MMN | 112.5 [18.4] | 0.23 (-2.86 to 3.32) | 0.88 |  |
| **Socio-emotional** |  |  |  |  |
| Daily IFA | 81.2 [10.5] | Reference |  | 0.03 |
| Twice weekly IFA | 81.9 [11.9] | 0.72 (-1.35 to 2.79) | 0.49 |  |
| Twice weekly MMN | 82.0 [11.5] | 0.87 (-0.71 to 2.45) | 0.28 |  |
| **General adaptive** |  |  |  |  |
| Daily IFA | 97.2 [9.9] | Reference |  | 0.02 |
| Twice weekly IFA | 97.4 [9.6] | 0.22 (-1.51 to 1.95) | 0.80 |  |
| Twice weekly MMN | 97.7 [10.1] | 0.51 (-0.86 to 1.87) | 0.46 |  |

*^1^* Model adjusted for clustering

CI - Confidence Interval

ICC- Intra-cluster Correlation Coefficient

IFA= Iron folic acid

MMN= Multiple micronutrients
