# Supplementary material for: The Effect of Intermittent Antenatal Iron Supplementation on Maternal and Infant Outcomes in Rural Viet Nam: A Cluster Randomised Trial
Source: PLoS Med. 2013 Jun 18;10(6):e1001470. doi: 10.1371/journal.pmed.1001470 (PMC3708703; doi:10.1371/journal.pmed.1001470)
Supplement: Text S3 — Ethics approval. (PDF) [file pmed.1001470.s003.pdf]

PO Royal Melbourne Hospital
Parkville Victoria 3050
Telephone 61 3 9342 8530
Facsimile 61 3 9342 8548
Email: research@mh.org.au
Website: http://research.mh.org.au
ABN 73 802 706 972

MELBOURNE HEALTH

OFFICE FOR RESEARCH

MELBOURNE HEALTH HUMAN RESEARCH ETHICS COMMITTEE - ETHICAL APPROVAL OF A RESEARCH PROJECT

Dr Beverley Biggs
The University of Melbourne Department of Medicine
The Royal Melbourne Hospital
C/- The Post Office
PARKVILLE VIC 3050

6th May 2010

Dear Dr Biggs

MH Project Number: 2010.061

Project Title: Does Weekly Iron Supplementation increase iron uptake in pregnant women and improve maternal and infant health?

HREC Approval Date: 6th May 2010 **HREC Approval Expiry**: 6th May 2013

I am pleased to advise that the above project has received ethical approval. This approval is valid for 3 years from the HREC approval date.

Participating Sites:

- *Department of Medicine University of Melbourne*

Approved Documents:

- Study Protocol dated 18th February 2010

Please note: You cannot commence this study until you have completed all the requirements of the Site Specific Assessment and have received the "Approval to Conduct a Research Project at Melbourne Health" certificate.

In order to comply with the National Statement on Ethical Conduct in Human Research 2007, Guidelines for Good Clinical Research Practice and Melbourne Health Research Policies and Guidelines you are required to:

- Submit a copy of this letter to the Radiation Safety Officer (RSO) at Melbourne Health, for addition of the project to the Licence for Research Involving Human Volunteers held by the Department of Human Services Radiation Safety Section Radiation Safety Licence (if your project involves exposure to ionising radiation). Note: You cannot commence the project until you have received notification from the RSO that the project has been added to the Licence;
- Notify the HREC of the actual start date of the project;
- Submit to the HREC for approval any proposed amendments to the project including any proposed changes to the Protocol, Participant Information and Consent Form/s and the Investigator Brochure;
- Notify the HREC of any adverse events in accordance with the Melbourne Health *Guidelines for Monitoring and Reporting of Safety in Clinical Trials Involving Therapeutic Products and Other Clinical Research, July 2009;*
- Notify the HREC of any unforseen events;

The Melbourne Health HREC operates and is constituted in accordance with the National Statement on Ethical Conduct in Human Research 2007.

- Notify the HREC of your inability to continue as Principal Investigator or any other change in research personnel involved in the project;
- Notify the HREC of any proposed extension to the duration of the project, past the above stated approval date; and
- Notify the HREC if a decision is taken to end the study prior to the expected date of completion or failure to commence the study within 12 months of the HREC approval date;
- Notify the HREC of any other matters which may impact the conduct of the project.

Reporting

You are required to submit to the HREC:

- An Annual Progress Report every 12 months for the duration of the project. This report is due on the anniversary of HREC approval; and
- A comprehensive Final Report upon completion of the project.

The HREC may conduct an audit of the project at any time.

Please refer to the Office for Research website to access forms such as the Amendment Form, Annual Report/Final Report Form, Guidelines for Monitoring and Reporting of Safety in Clinical Trials Guidelines and Adverse Event Report Forms, and other information and news concerning research at Melbourne Health: http://www.mh.org.au/www/342/1001127/displayarticle/1001352.html

A list of those HREC members present at the review of this project can be obtained from the above website.

Yours sincerely,

Ms. Angela Gray
Manager, Melbourne Health Human Research Ethics Committee
Ph: 9342 3006
E-mail: angela.gray@mh.org.au

The Melbourne Health HREC operates and is constituted in accordance with the National Statement on Ethical Conduct in Human Research 2007.

PO Royal Melbourne Hospital
Parkville Victoria 3050
Telephone 61 3 9342 8530
Facsimile 61 3 9342 8548
Email: research@mh.org.au
Website: http://research.mh.org.au
ABN 73 802 706 972

MELBOURNE HEALTH

OFFICE FOR RESEARCH

APPROVAL TO CONDUCT A RESEARCH PROJECT AT MELBOURNE HEALTH
SITE SPECIFIC ASSESSMENT (SSA) AUTHORISATION

Dr Beverley Biggs
The University of Melbourne Department of Medicine
The Royal Melbourne Hospital
C/- The Post Office
PARKVILLE VIC 3050

6th May 2010

Dear Dr Biggs

HREC Reference Number: *N/A* **MH Project Number:** 2010.061

Project Title: Does Weekly Iron Supplementation increase iron uptake in pregnant women and improve maternal and infant health?

SSA Approval Date: 6th May 2010

HREC Approval Date: 6th May 2010 **HREC Approval Expiry**: 6th May 2013

I am pleased to advise that the above project is approved to be conducted at Melbourne Health. This approval is subject to compliance with any conditions imposed by the reviewing HREC.

Approved Documents:

- Melbourne Health Approval Letter dated 6th May 2010
- Study Protocol dated 18th February 2010

You are required to notify the Office for Research of:

1. The actual start date of the project at Melbourne Health.
2. Any amendments to the project after these have been approved by the reviewing HREC.
3. Any adverse events involving patients of Melbourne Health, in accordance with the Melbourne Health *Guidelines for Monitoring and Reporting of Safety in Clinical Trials Involving Therapeutic Products and Other Clinical Research, July 2009.*
4. Any unforseen events.
5. Any changes to the indemnity, insurance arrangements or Clinical Trial Research Agreement for this project. This includes changes to the project budget or other changes which may have financial or other resource implications for Melbourne Health.
6. Your inability to continue as Principal Investigator or any other change in research personnel involved in the project.
7. Any proposed extension to the duration of the project, which has been approved by the Reviewing HREC.
8. Any other matters which may impact the conduct of the project at Melbourne Health.

You are also required to submit to the Office for Research:

1. A copy of the TGA acknowledgement letter in respect of the CTN notification (if applicable).
2. An Annual Progress Report every 12 months for the duration of the project. This report is due on the anniversary of HREC approval.

3. A comprehensive Final Report upon completion of the project.

The Office for Research may conduct an audit of the project at any time.

Please refer to the Office for Research website to access forms such as the Amendment Form, Annual Report/Final Report Form, Guidelines for Monitoring and Reporting of Safety in Clinical Trials Guidelines and Adverse Event Report Forms, and other information and news concerning research at Melbourne Health: http://www.mh.org.au/www/342/1001127/displayarticle/1001352.html

Yours sincerely,

pp HREC Manager
Dr Angela Watt
Manager Office for Research
